# Record‐High Latent Heat, Ultra‐Fast Relaxation and Closed‐Loop Recycling Double‐Brush Polymer Networks for Self‐Adaptive Thermal Interface Management

**DOI:** 10.1002/advs.202521482

**Published:** 2025-12-23

**Authors:** Qiguang Liu, Yanyun Li, Zhenghao Wu, Junjie Cheng, Chang Jing, Jue Cheng, Jiahao Ma, Junying Zhang

**Affiliations:** ^1^ College of Materials Science and Engineering Beijing University of Chemical Technology Beijing China

**Keywords:** bottlebrush polymer, covalent adaptable networks, phase change materials, thermal interface materials, ultra‐high latent heat

## Abstract

In the era of artificial intelligence (AI)‐driven high‐performance computing, phase change materials (PCMs) are critical for high‐flux thermal management. PCMs are evolving toward high enthalpy, low interfacial thermal resistance (ITR), and high reliability. Herein, we design double‐brush phase‐change polymer (PVBS‐TMC_n_) crosslinked by B─O─B and Si─O─B dynamic bonds, characterized by the ultra‐fast relaxation time of 0.8 s under 80°C and closed‐loop cycling. This architecture enhances the content of phase‐change units for elevated theoretical enthalpy, while inherent multiple dynamic bonds and ultra‐low entanglement minimize enthalpy loss, resulting in a record enthalpy of 240.7 J·g^−1^. Furthermore, a composite of flexibility PVBS‐TMC_14/24_ and graphene foam films (PVBS‐TMC/GF) is fabricated as thermal interface materials using a stacking‐cutting strategy, which self‐adaptively modulates low‐ITR in response to temperature, owing to phase transition properties, ultra‐low modulus, and adaptive filling capability of dynamic polymer matrix. PVBS‐TMC/GF significantly generates better thermal management efficiency compared to commercial products. The topology design of double‐brush polymer dynamic networks and interfacial contact mechanisms provide fundamental insights for developing phase‐change adaptive materials and advancing thermal management.

## Graphical abstract

By grafting dense long double‐brush chains, we synthesized bottlebrush networks with high theoretical enthalpy. Their low entanglement, combined with dynamic bonds, promotes crystalline domain reorganization and minimizes enthalpy loss, achieving a record enthalpy of 240.7 J·g^−1^. The low modulus of bottlebrush networks and interfacial reorganization ability of dynamic bonds enhance contact and micro‐void infiltration in TIMs, enabling self‐adaptive ITR under thermal stimulation.

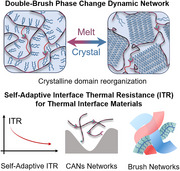

## 1 Introduction

As semiconductor processes approach their physical limits, high integration packaging in AI era intensifies chip thermal load [1], necessitating advanced thermal management systems to meet escalating heat flux demands [2]. PCMs regulate temperature fluctuations through latent heat mode during reversible phase transitions [3], proving indispensable for thermal management [4]. Inevitably, PCMs demand structural stability to ensure device reliability [5], exceptional latent heat (enthalpy change, Δ*H*) to mitigate thermal shock [6], and optimized interfacial contact to minimize ITR for efficient heat dissipation [7]. Consequently, the development of integrated PCMs with superior Δ*H*, outstanding ITR, and high reliability, proves imperative in the AI‐driven era [8].

Polymeric solid‐solid PCMs are constructed from phase‐change polymer networks (PCPNs), where phase‐change units are chemically anchored within networks, demonstrating exceptional stability albeit with suppressed Δ*H*, hardly exceeds 160 J·g^−1^ [9, 10]. The Gibbs free energy relationship of phase transition process indicates that Δ*H* equals TΔ*S* (entropy change) under reversible condition [11], where Δ*S* is governed by Boltzmann relation [12]. This fundamental highlight that the conformational complexity of phase‐change units directly determines theoretical Δ*H* ^[^ 13]. In existing PCPNs, the enhancement of conformational complexity is achieved by increasing the content of phase‐change units [14]. In addition, chain entanglements and steric hindrance limit chain mobility, so phase‐change units are not fully incorporated into the crystalline domains, which suppresses crystal formation and results in a reduction of Δ*H* (Δ*H*_loss_) [15, 16, 17].

The pursuit of enhancing the content of phase‐change units within PCPNs to enhance Δ*H* through topological design remains a central research focus [18]. However, the content of phase‐change units has reached its limit. Moreover, such high loading often creates significant molecular entanglement, presenting barriers to crystalline formation and growth [15, 19]. During the induction period of the phase transition process, phase‐change units first undergo disentanglement before entropy‐driven arrangement into crystalline lattices, which increase Δ*H*_loss_ [20]. The lattice dimensions expand with increasing phase‐change chain incorporation, where the larger crystalline domains determine higher Δ*H* [21]. Additionally, PCPNs employ permanent crosslinked networks for reliability, such irreversible networks restrict mobile crystalline segments, also causing Δ*H*_loss_ [22]. Consequently, incorporating more phase‐change units into PCPNs enhances theoretical Δ*H*, while designing low‐entanglement topological architectures with spatially reconfigurable networks dramatically diminishes Δ*H*_loss_, enabling revolutionary advances in enthalpy.

PCMs have become widely adopted in thermal interface materials (TIMs), where they soften upon gradual heating and conform to interfaces, reducing ITR while buffering temperature peaks, thereby enhancing thermal management efficiency [4, 5]. Composites incorporating paraffin represent the most typical phase‐change thermal interface materials (PCM‐TIMs). However, these suffer from poor shape stability and leakage tendencies [18]. Moreover, to address the intrinsic problem of low thermal conductivity of PCMs [23], advancements have relied on physically blending high filler loadings such as carbon materials [24, 25], boron nitride [26], liquid metal [27], and other thermally conductive fillers [28, 29]. Although high filler loadings enhance thermal conductivity, the resultant modulus increasing and poor interfacial compatibility exacerbate ITR [7, 30]. Therefore, it is still a great challenge to achieve integrated PCM‐TIMs with reliability, flexibility, high thermal conductivity, and minimized ITR.

**FIGURE 1:**
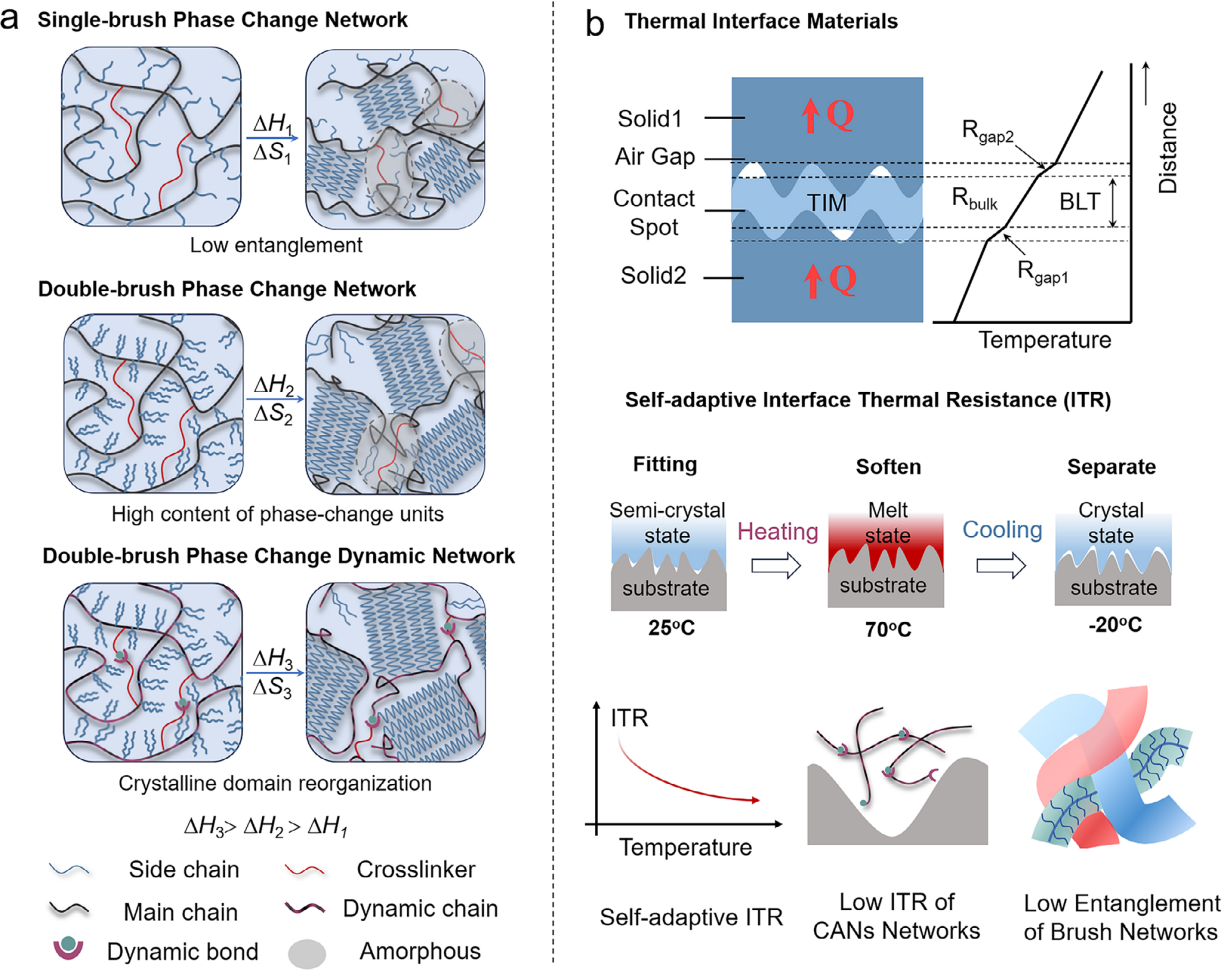
The design concept of this work. (a) Topological design of double‐brush dynamic PCPNs. (b) Schematic diagram of TIMs based on double‐brush dynamic PCPNs and self‐adaptive ITR mechanism.

Bottlebrush polymer networks exhibit densely grafted side‐chain, low entanglement, and reducing modulus [31, 32, 33, 34, 35, 36, 37, 38], with their distinctive topology proving effective for enhancing Δ*H* [39, 40]. Covalent adaptable networks (CANs), formed by incorporating dynamic covalent bonds into polymer networks, permit topological rearrangement to achieve self‐healing, closed‐loop recyclability [41, 42, 43], and foreseeable structural evolution. Consequently, we rationally infer that increasing side‐chain grafting density elevates theoretical Δ*H* [20], while introducing dynamic bonds of bottlebrush networks further promotes crystalline domain reorganization [44] beyond low entanglement, can achieve ultra‐low Δ*H*_loss_ (Figure 1a). Simultaneously, the low modulus of bottlebrush networks combined with the interfacial reorganization capability of dynamic bonds enable interfacial contact and facilitate micro‐void infiltration in TIMs, enabling self‐adaptive ITR under thermal stimulation (Figure 1b) [4, 7, 45].

Herein, the key to achieve our concept is designing double‐brush dynamic PCPNs to maximally increase density of phase‐change units, decrease entanglement and adaptively rearrange, achieving record enthalpy PVBS‐TMC_32_ (Δ*H* up to 240.7 J·g^−1^, 53.3% higher than the published value [9]), while maintaining ultra‐low modulus (storage modulus <10 kPa) in molten state. The introduction of dynamic Si─O─B and B─O─B bonds within both the backbone (PVBS) and crosslinker (TSH) enables rapid network rearrangement (0.8 s relaxation time under 80°C), alleviating segment migration barriers during phase transition. Concurrently, these dynamic bonds impart self‐adaptive characteristics and enable catalyst‐free closed‐loop recycling. Furthermore, we also investigated the structure‐activity relationships of these dynamic networks with different chain lengths. Moreover, a stacking‐cutting strategy was employed to composite the PVBS‐TMC_14/24_ with graphene foam films (GF), producing layered TIMs, where the GF layers are aligned parallel to the heat flow direction. This resulted in a significantly reduced adaptive ITR (35.7 K·mm^2^·W^−1^ at 80°C and 10 N) and a high thermal conductivity of 55.5 W·m^−1^·K^−1^. When applied to computer Central Process Unit (CPU), this composite achieves a 10°C–15°C reduction in steady‐state temperature and an 8°C–10°C reduction in peak temperature compared to commercial products. This composite provides a critical solution for high‐flux thermal management.

## 2 Results and Discussion

### 2.1 Design and Synthesis of Main Chain, Side Chain and Crosslinker of Double‐Brush Dynamic PCPNs

**FIGURE 2:**
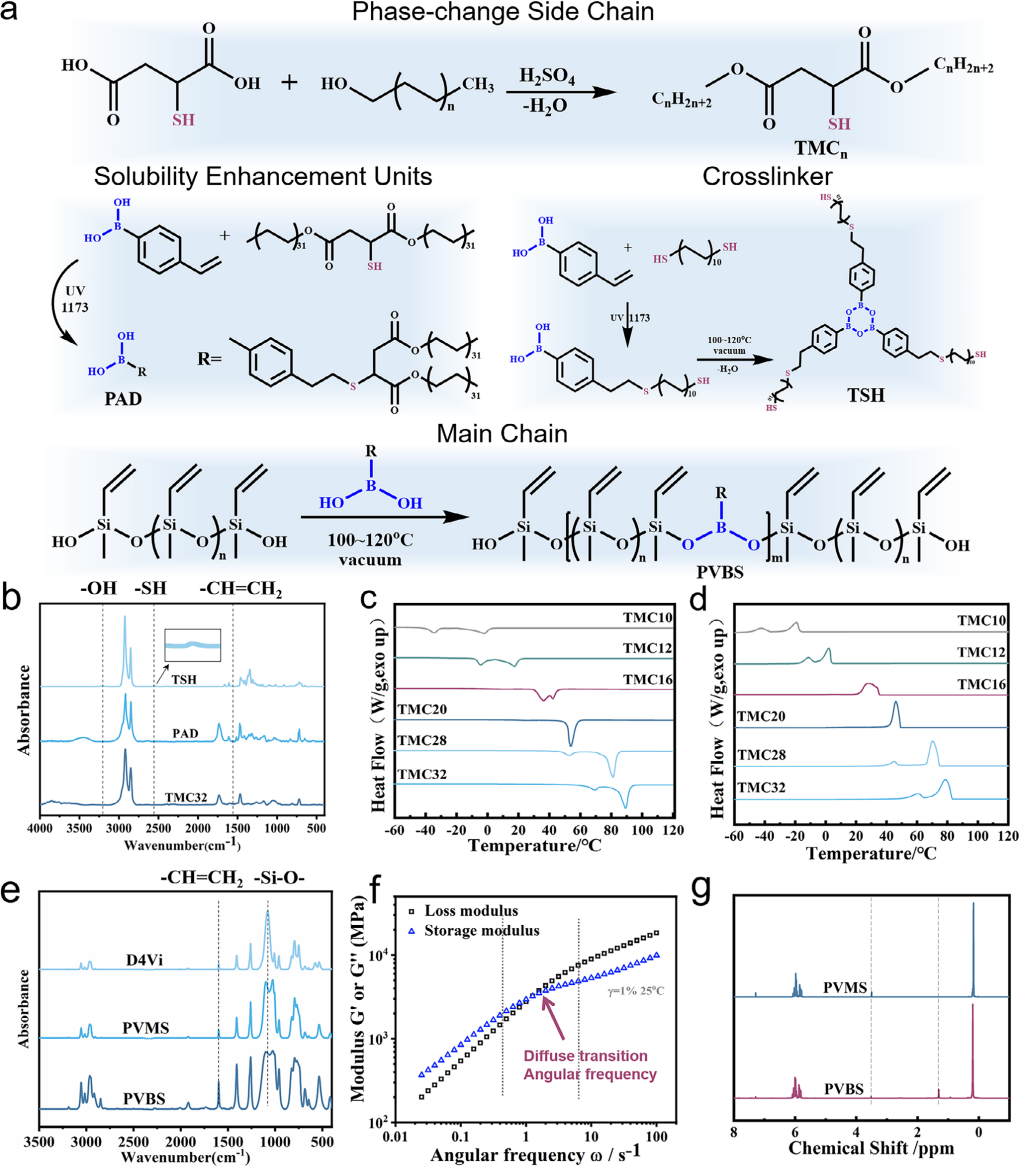
Synthesis and characterization of main chain, side chain, and crosslinker. (a) The route of reaction of main chain, side chain, and crosslinker. (b) FT‐IR spectra of TMC_32_, PAD, and TSH. (c‐d) DSC curves of TMC_n_. (e) FT‐IR spectra of D4Vi, PVMS, and PVBS. (f) Shear thickening behavior of PVBS. (g) ^1^H NMR spectra of PVMS and PVBS.

In the process of synthesis reactive phase‐change units, thiomalic acid (TMC) was selected as the sulfhydryl reagent to undergo esterification with a series of 1‐n‐alkanol derivatives, and the resulting esterification products, are designated as TMC_n_ (n = 10, 12, 14, 16, 18, 20, 22, 24, 28, and 32) (Figure 2a). Fourier transform‐infrared spectroscopy (FT‐IR) spectroscopy revealed the near‐complete disappearance of the hydroxyl absorption band in the 3000–3500 cm^−1^ region (Figure 2b; Figure S1a). In the nuclear magnetic resonance (NMR) (Figure S1b,c) analysis, the carboxyl carbon peaks of TMC (δ = 172.7 and 174.7 ppm) in ^13^C NMR underwent a downfield shift to δ = 170.4 and 172.4 ppm upon ester bond formation post‐reaction, consistent with the electronic redistribution induced by esterification (Figure S1d). Due to the low polarity and low content of thiol groups, the characteristic peak of thiol groups (2550–2600 cm^−1^) was difficult to observe (Figure S1a). Additionally, TMC_32_ and D4Vi are designed as a model (Figure S2a). The disappearance of vinyl peak in FT‐IR spectrum (Figure S2b) and ^1^H NMR spectrum (Figure S2c,d) demonstrate the reactivity of TMC_n_.

Differential scanning calorimetry (DSC) analysis (Figure 2c,d; Figure S3a,b) demonstrated that the Δ*H* of TMC_n_ increased from 129.6 J·g^−1^ (TMC_10_) to 264.0 J·g^−1^ (TMC_32_), with a broad melting temperature range spanning −2.5°C to 88.0°C, establishing a robust foundation for high‐enthalpy PCPNs. X‐ray diffraction (XRD) patterns (Figure S3c) exhibited two characteristic peaks at 2θ = 21.5° and 23.8° (*d* = 4.1 and 3.7 Å calculated by Bragge formula), accompanied by weak secondary peaks at 30.0°, 36.2°, and 40.6° (*d* = 3.0, 2.5, and 2.2 Å), collectively indicating a crystalline structure analogous to long‐chain alkanes. Notably, as the alkyl chain length increased, crystallinity improved significantly, while shorter chains (TMC_14_ and TMC_16_) formed incomplete crystalline structures at ambient temperature due to their lower melting points, manifesting partial secondary crystallization peaks. Thermogravimetric analysis (TGA) further corroborated that shorter‐chain derivatives underwent decomposition more readily, whereas longer chains (such as TMC_32_) exhibited enhanced thermal stability (Figure S3d,e).

Polyvinylborosiloxane (PVBS), as main chain, was synthesized via the reaction of polyvinylmethylsiloxane (PVMS) with boron‐containing compounds. Initial experiments revealed phase separation between PVBS, comprising methylboronic or 4‐vinylphenylboronic acid (VPBA), and TMC_n_ (Figure S4a), necessitating solubility enhancement. A strategic approach employing click chemistry between VPBA and TMC_32_ yielded phenylboronic acid‐alkane derivatives (PAD) (Figure 2a). In the FT‐IR spectra of PAD (Figure S5a), the vinyl peak at 1553cm^−1^ of VPBA disappeared. The vinyl hydrogen at chemical shifts of δ = 6.75, 5.83, and 5.27 ppm disappeared in the ^1^H NMR spectrum of the PAD (Figure S5b), proving the successful synthesis of PAD. Subsequent vacuum‐assisted high‐temperature reactions between B─OH and Si─OH groups enabled the covalent incorporation of long alkyl chains into the PVBS backbone (Figure 2a), effectively improving compatibility (Figure S4b). FT‐IR analysis of PVBS (Figure 2e) showed peak broadening at 1100 cm^−1^, indicating successful grafting of phenylboronic acid‐alkane derivatives onto the polymer backbone, attributed to supramolecular interactions between electron‐deficient boron centers and lone electro n pairs of Si─O bonds altering vibrational states [46]. This unique boron‐oxygen supramolecular interaction not only endowed PVMS with distinctive shear‐thickening behavior (Figure 2f) but also imparted exceptional damping properties (Figure S7a) across a broad frequency range (0.001–100 s^−1^).

In NMR spectrum of PVBS, wherein the peak at 1.1 ppm in the ^1^H NMR spectrum (Figure 2g) and the peaks around 30.3 ppm in the ^13^C NMR spectrum (Figure S6a) correspond to the chemical shifts of hydrogens on the carbon chain in the phenylboronic acid derivative, indicating the successful integration of PDA. The decrease in the area at the peak of 3.33 ppm in the ^1^H NMR spectrum of the hydroxyl group indicates the occurrence of dehydration condensation. Due to the quadrupolar effect of ^11^B, a broad peak composed of three subpeaks was observed in the ^11^B NMR spectrum (Figure S6b). Due to the residual reactants and boron‐oxygen attractive, unequal triple peaks emerged [47]. This was consistent with the GPC results of PVBS (Table S1). After dehydration condensation, the molecular weight of PVBS is approximately 2.2 times that of PVMS, and a wider distribution (*Đ* = 2.99) was observed compared to PVMS (*Đ* = 1.74). Due to the low addition amount of the boron derivative and the low natural abundance and gyromagnetic ratio of ^29^Si, the influence of the boron derivative on the ^29^Si NMR signals was submerged in a noisy background (Figure S6c). Additionally, the viscosity of PVMS and PVBS were measured (Figure S7b). The above results proved the successful synthesis of PVBS.

To endow the PCPNs with recyclability, we designed a novel trithiol crosslinker (TSH) through rational molecular engineering. As illustrated in Figure 2a, the synthesis involved reacting VPBA with excess 1,10‐decanedithiol (DDT) to yield a mercapto‐functionalized phenylboronic acid derivative. FT‐IR spectroscopy (Figure S8a) demonstrated the successful synthesis through the disappearance of the vinyl stretching vibration at 1553 cm^−1^ and the elimination of hydroxyl absorption in the 3000–3500 cm^−1^ range. Meanwhile, ^1^H NMR analysis confirmed complete consumption of the vinyl protons from the VPBA (δ = 6.75, 5.83, and 5.27 ppm) (Figure S8b). The increasing of ^11^B NMR at −4.13 ppm confirmed the expected boroxine ring structure (Figure S8c).

Owing to the inherent long alkyl chains, the resultant main chain, side chain, and crosslinker exhibited distinct phase transition temperatures and Δ*H*, with specific thermal characteristics systematically documented in Table S2.

### 2.2 Synthesis and Properties of Double‐Brush PCPNs (PVBS‐TMC_n_)

**FIGURE 3:**
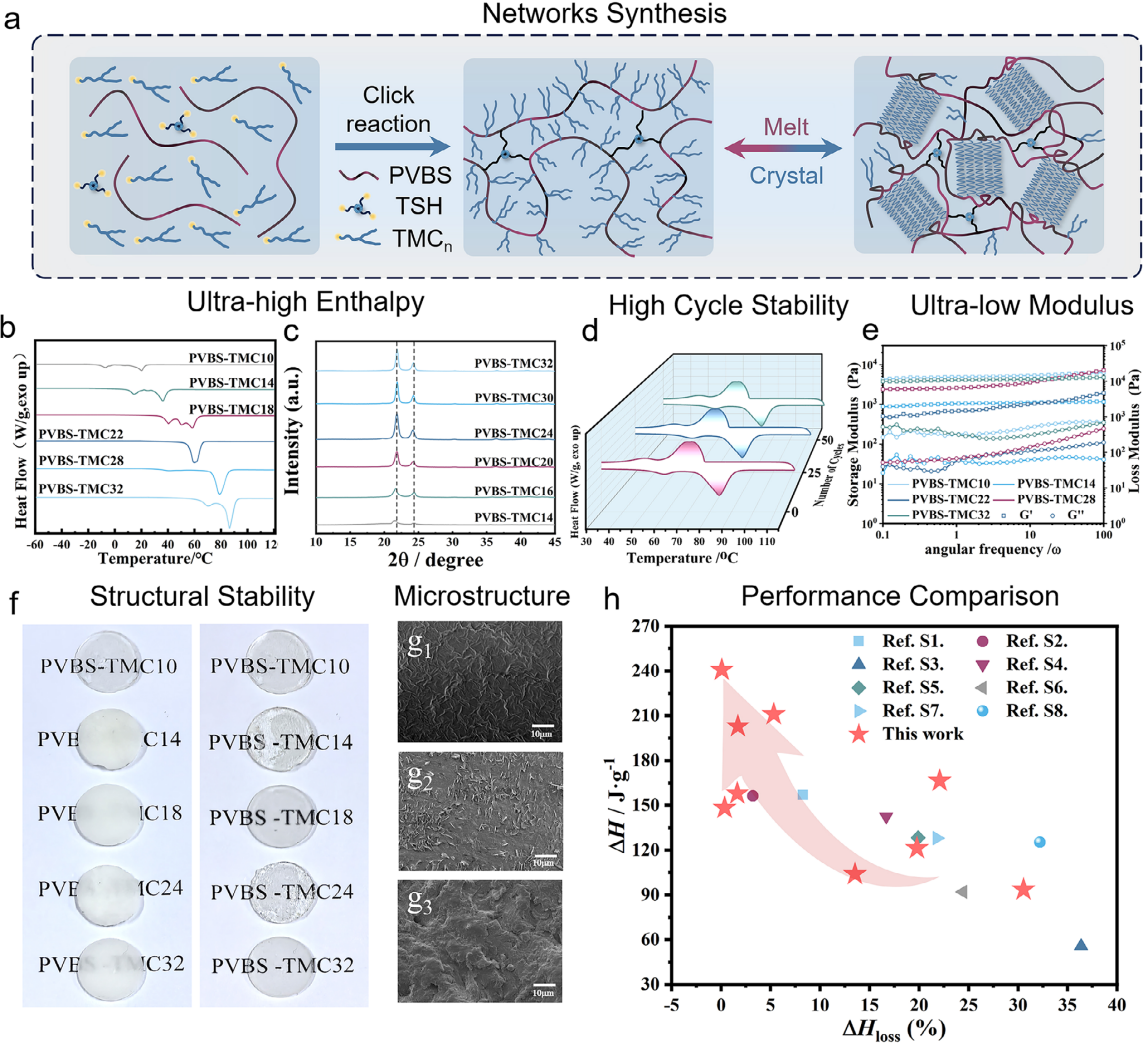
Preparation of PVBS‐TMCn networks and their performance characterization. (a) Schematic diagram of the synthesis and phase transition behavior of PVBS‐TMC_n_ networks. (b) The melting process of DSC and (c) XRD of PVBS‐TMC_n_. (d) DSC diagram of PVBS‐TMC_32_ during multiple heating and cooling cycle. (e) Rheology curves, (f) Visual picture, and (g) SEM image of PVBS‐TMC_n_. (h) Comparisons of Δ*H* and Δ*H*_loss_ of PVBS‐TMC_n_ with other reported works.

Through compatibilization optimization, we achieve efficient in‐situ curing and bulk crosslinking, enabling large‐scale preparation and application. Then, high‐enthalpy and closed‐loop recyclability double‐brush polyborosiloxane phase‐change networks (PVBS‐TMC_n_) (n = 10, 12, 14, 16, 18, 20, 22, 24, 28, and 32) were successfully fabricated via a facile click reaction (Figure 3a). FT‐IR spectra of PVBS‐TMC_32_ as typical sample confirmed the disappearance of the vinyl group absorption band at 1597 cm^−1^, validating the completion of the thiol‐ene click reaction (Figure S9a).

DSC curves of PVBS‐TMC_n_ indicated phase transition behaviors with varying grafted chain lengths (Figure 3b). The melting temperatures (*T*_m_) of PVBS‐TMC_n_ spanned from −29.4°C to 83.4°C, accompanied by melting enthalpies (Δ*H*_m_) ranging from 35.5 to 240.7 J·g^−1^ (Figure S9b). Similarly, crystallization processes (Figure S9c) exhibited crystallization temperatures (*T*_f_) of PVBS‐TMC_n_ between −19.3°C and 79.7°C, with crystallization enthalpies (Δ*H*_f_) escalating from 139.2 to 240.4 J·g^−1^. All thermal data have been systematically documented in Table S2. Wherein, PVBS‐TMC_32_ exhibited ultra‐high Δ*H* (240.7 ± 1.8 J·g^−1^) comparing with other work (Figure 3h; Table S3).

XRD analysis (Figure 3c) revealed characteristic peaks at 2θ = 21.9° and 24.3°, corresponding to interplanar spacings of d = 4.1 and 3.7 Å calculated via Bragg formula. Longer alkyl chains intensified peak intensities without altering crystal structure, belonging to cross‐lamellar structure [48]. Shorter chains exhibited peak shifts toward lower angles, reflecting expanded lattice spacing and looser packing, further validating the strategy of augmenting phase‐change units to boost crystallinity and Δ*H*. Cyclic DSC testing demonstrated excellent thermal stability over 50 cycles for most PVBS‐TMC_n_ (Figure 3d; Figure S10a–i and Table S4), with only PVBS‐TMC_12_ and PVBS‐TMC_14_ showing moderate Δ*H* degradation.Predicting these results to 1000 cycles, the enthalpy of PVBS‐TMC_32_ is estimated to remain at approximately 207.1 J·g^−1^ under the assumption of linear attenuation, demonstrating exceptional robustness. Furthermore, subjecting PVBS‐TMC_32_ to prolonged thermal aging at 100(C for 168 h (7 days) preserve 217.5 J·g^−1^ of Δ*H* (Figure S10j), with no observed macroscopic cracking or significant performance degradation.TGA profiles (Figure S11a,b) revealed progressive improvement of thermal stability with alkyl chain elongation, with PVBS‐TMC_32_ maintaining stability up to ∼300°C. Critically, no decomposition occurred below 150°C, ensuring operational reliability under intended thermal management conditions.

Dynamic mechanical analysis (DMA) of PVBS‐TMC_n_ showed the storage modulus initially increases then decreases at low temperatures with elongating side‐chain length, due to enhanced crystal size (Figure S12a). The loss modulus curve (Figure S12b) displayed characteristic peaks corresponding to sequential chain segment activation upon heating. Three distinct relaxation processes are identified in the tanδ plot (Figure S12c,d), which respectively correspond backbone motion, carbon chains movement adjacent to the siloxane backbone, and alkyl chain mobility prior to melting [39]. The above data proved that the network can still perform slight movement below the melting temperature, supporting for the rearrangement of dynamic bonds. Additionally, we conducted an extra cyclic DMA test from room temperature to melting temperature to ensure the reversibility of phase transformation during the cycle (Figure S13b,c), wherein the heating and cooling processes cannot completely overlap due to supercooling, which is also observed in DSC curve (Figure S3a,b). Notably, PVBS‐TMC_12_ exhibited characteristics of damping materials across −80°C to −10°C, stemming from enhanced boron‐oxygen attractive and exchanged hydroxyl under the reduced steric hindrance from short alkyl side chains, generating multiple overlapping relaxation processes [49].

Oscillatory rheology at 90°C (above melting point, Figure 3e) revealed ultra‐low storage modulus (<10 kPa), confirming excellent molten‐state softness. The viscoelastic response overall revealed soft gel behavior [50] and followed power‐law frequency dependence (*G*''∼*G*'∼ωⁿ) in 1–100 rad/s [51]. Contrary to conventional bottlebrush expectations where elongated side chains reduce entanglement through decreased hydrodynamic volume, PVBS‐TMC_10_ and PVBS‐TMC_32_ paradoxically showed comparable storage modulus. Meanwhile, power‐law exponents n for *G*''‐frequency relationships, such as 0.23 (PVBS‐TMC_10_), 0.23 (PVBS‐TMC_12_), 0.27 (PVBS‐TMC_14_), 0.25 (PVBS‐TMC_18_), 0.26 (PVBS‐TMC_20_), 0.27 (PVBS‐TMC_22_), 0.32 (PVBS‐TMC_28_), 0.47 (PVBS‐TMC_30_), and 0.33 (PVBS‐TMC_32_), indicate the entanglement first decrease and then increase [40]. This anomaly arises from partial entanglement formation in longer chains, theoretical calculations based on freely rotating polyethylene chains yielded Kuhn lengths of 1.2 for PVBS‐TMC_10_, which dilute entanglement versus 4.0 for PVBS‐TMC_32_, which enable measurable entanglement [52]. The densely long grafted double‐brush architecture results in forced chain entanglement while maintaining a low entanglement level. This ascending trend in n aligns with the calculated Kuhn lengths and ensures the reduction of non‐phase‐change energy loss, which is conducive to achieving low Δ*H*_loss_.

The macroscopic characteristics of fabricated PCPNs are illustrated in Figure 3f. At 25°C, PVBS‐TMC_10_ exist in a transparent molten state, while others PVBS‐TMC_n_ (n = 12–32) remain opaque solids. Upon heating to 90°C, all PVBS‐TMC_n_ transition to fully transparent molten states, demonstrating exceptional substrate conformality by tightly adhering to paper surfaces without leakage (Figure S14). Crucially, no residual material was observed on the paper after removal of sample, confirming the intrinsic solid‐solid phase transition behavior and structural stability. The Scanning electron microscopy (SEM) images of PVBS‐TMC_n_, show soft and elastic PVBS‐TMC_10_ gradually transited to hard and brittle PVBS‐TMC_32_ with increasing crystalline (Figure 3g_1_‐g_3_; Figure S15a–h). In addition, SEM‐EDS shows that various elements are uniformly distributed in PVBS‐TMC_n_ without obvious macroscopic or microscopic phase separation (Figure S15i).

### 2.3 Ultra‐Fast Relaxation and Closed‐Loop Recyclability of PVBS‐TMC_n_

**FIGURE 4:**
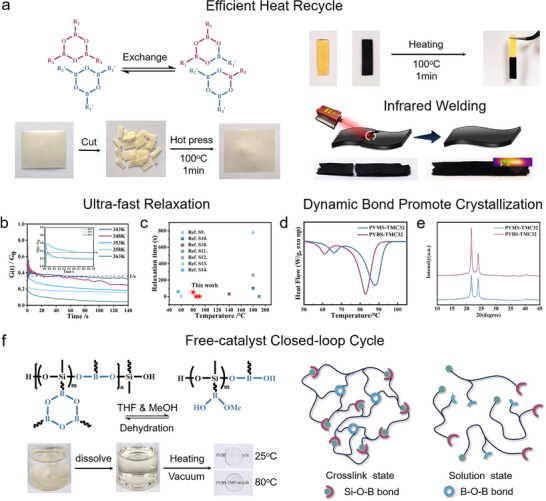
The dynamic network characteristics of PVBS‐TMCn and its recyclability. (a) Schematic diagram of dynamic bonds exchange, and visual diagram of hot‐press, and infrared welding schematic diagram, and physical welding effect of PVBS‐TMC/CNTs. (b) Stress relaxation of PVBS‐TMC_n_ and (c) comparison of relaxation time and temperature between PVBS‐TMC_n_ and other reported dynamic networks. (d) DSC and (e) XRD curves of PVMS‐TMC_32_ and PVBS‐TMC_32_. (f) Solvent closed‐loop recycling of PVBS‐TMC_n_.

We incorporated the dynamic bond both in backbone and crosslinker. In fact, PVBS‐TMC_n_ is a kind of CANs, which causing segment ultra‐fast relaxation. Above the melting temperature, the rapid reconfiguration of networks occurred via dynamic exchange of B─O─B bonds (Figure 4a), and Si─O─B bond rearrangement (Figure S16a) in PVBS‐TMC_n_ networks, enabling complete hot‐press recycling within 1 min at 100°C (Figure 4a). FT‐IR analysis under a series of temperature below the *T*_m_ (Figure S16b) shows the B─O bond transitions from the C_2v_ point group to the C_3h_ point group [47], demonstrating molecular mobility under crystalline state, with evidence corroborated by the b‐transition observed at low‐temperature range by DMA (Figure S12c,d), collectively providing evidence for backbone unit (Si─O─Si and Si─O─B) motion of networks at crystalline state. Simultaneously, without external pressure, by aligning and heating to 100°C, the two splines can also be combined macroscopically and remain intact without falling off (Figure 4a).

Stress relaxation measurements of PVBS‐TMC_32_ via rheometer (Figure 4b; Figure S16c) revealed remarkable dynamics. Characteristic relaxation times (t) of 1/e stress decay decreased from 51.9 s (70°C) to 0.8 s (80°C) and 0.056 s (90°C). Arrhenius analysis yielded an activation energy Δ*E_a_* = 403.6 kJ·mol^−1^ (R^2^ = 0.9366). To decouple side‐chain effects, a side‐chain‐free dynamic linear networks was prepared, yielding stress relaxation profiles and linear fitting results (Figure S16d,e) with Δ*E_a_* = 23.5 kJ·mol^−1^ (R^2^ = 0.9983). The exceptionally high activation energy barrier of PVBS‐TMC_32_ was attributed to constrain motion from partially molten crystalline domains and densely packed side chains. These values indicate ultra‐fast relaxation of PVBS‐TMC_n_ compared with the published works (Figure 4c; Table S5).

Therefore, the breakthrough of Δ*H* in PCPNs lies in the ultra‐high phase‐change units content enabled by the double‐brush bottlebrush architecture, which provides exceptionally high theoretical Δ*H*. Simultaneously, the low entanglement induced by the bottlebrush structure, combined with the ultra‐fast relaxation of dynamic bonds, achieves ultra‐low Δ*H*_loss_. Specifically, besides entanglement‐induced Δ*H*_loss_, steric hindrance, caused by the attachment of crystalline units to the backbone, constitutes another major cause of Δ*H*_loss_. Ultra‐fast relaxation mitigates this steric hindrance‐induced Δ*H*_loss_. For example, PVBS‐TMC_32_ exhibited ultra‐low Δ*H*_loss_ of 0.06% when calculated based on mass fraction, which can be attributed to the dynamic polyborosiloxane network architecture enables reorganization during crystallization. Relatively, for low *T*_m_ or *T*_f_ of short‐chain PVBS‐TMC_n_ (n = 10–24), insufficient dynamic bond exchange motion, which was also proved by relaxation behavior, to rearrange crystal lattice led to prominent Δ*H*_loss_. Unlike permanent networks or dynamic networks with high‐relaxation time, where rigid crosslinks hinder chain reorganization, the fast relaxation B─O─Si and B─O─B bond exchange promotes crystalline domain reorganization.

To validate the role of dynamic bonds in mitigating Δ*H*_loss_, PVMS‐TMC_32_ without dynamic bond was synthesized using PVMS as the backbone, DDT as crosslinkers, and TMC_32_ as side chains (Figure S17). Maintaining identical crosslinking density, this static network exhibited Δ*H*_loss_ of 8.43%, confirming that dynamic bond effectively reduces Δ*H*_loss_ (Figure 4d). Simultaneously, dynamic bonds to increased conformational entropy and facilitated extended crystalline domains, as corroborated by XRD crystallinity results in Figure 4e. After the incorporation of dynamic bonds, the crystallinity increased from 69.4% to 72.5%. Crystallization kinetics studies further supported this mechanism, that increasing the length of the chain reduces the bit hindrance of the network, leading to an increase in the activation energy (Figure S18a–c).

Similarly, dynamic bond exchange can change the phase transition temperature. Peculiarly, the *T_m_* of PVBS‐TMC_32_, PVBS‐TMC_30_, and PVBS‐TMC_28_ is lower than their pristine phase‐change units in Table S2, which is opposite to typical phase‐change behavior of grafting unit [39]. This phenomenon stemmed from the competition between dynamic bond dissociation temperature and *T*_m_. When the onset temperature for alkyl chain motion is reached, the dynamic bonds have already begun rapid rearrangement, significantly easing backbone constraints on phase‐change units and lowering *T*_m_.

Furthermore, incorporating 1 wt.% carbon nanotubes (CNTs) into PVBS‐TMC_22_ matrix named PVBS‐TMC/CNTs endowed infrared photothermal conversion capability via photon‐electron‐phonon interactions (Figure 4a). Under 808 nm laser irradiation (1 W), surface temperature rapidly reached 67°C within 30 s (Figure S19), triggering accelerated dynamic bond exchange that enabled macroscopic crack healing. This photothermal responsiveness facilitated infrared welding for constructing complex architectures, as exemplified by the laser‐welded star‐shaped pattern in Figure S20.

In fact, the B─O─B and Si─O─B bonds can be disrupted by hydroxyl groups in the network [53, 54]. Wherein, the trimeric boroxine ring decomposes into three phenylboronic acid molecules under hydroxyl attack, and Si─O─B is dissociated into B─OH and Si─OH. Considering the hydrophobic nature of PVBS‐TMC_n_ composed of organosilicon and alkyl chains, a mixed solvent of tetrahydrofuran (THF) and methyl alcohol (MeOH) was adopted, wherein THF for network swelling and MeOH for providing hydroxyl groups to dissociate networks. The densely packed alkyl chain structure exhibited solvent resistance at room temperature, while complete B─O─B and B─O─Si bond dissociation occurred within 3 h above *T*_m_. The obtained solution could be evaporated under heating and vacuum, followed by dehydration or dealcoholizing between molecular chains to reform crosslinked networks (Figure 4f). The dissolved sample (M_n_ = 3000 g/mol) exhibited a monomodal peak in GPC analysis (Figure S21), confirming complete dissolution and chemical reaction homogeneity. Concurrently, side‐chain‐induced backbone expansion diameter and decrease hydrodynamic volume, resulting in a substantially lower number‐average molecular weight compared to the initial polymer segment PVMS (M_n_ = 6300 g/mol). The reformed network maintained excellent thermal stability without leakage above *T*_m_. Post‐recycling characterization revealed minimal structural degradation. Solvent‐recycled samples (re‐PVBS‐TMC_32_‐D) showed 1.2°C depression of *T*_m_ with 3% Δ*H* reduction, while hot‐pressed counterparts (re‐PVBS‐TMC_32_‐H) exhibited 1.3°C depression of *T*_m_ and 7% Δ*H* reduction (Figure S22a). Compared with original PVBS‐TMC_32_, FT‐IR spectra (Figure S22b) confirmed structural integrity preservation after physical or chemical recycling. Above statistic proved that PVBS‐TMC32 can be recycled in a closed‐loop. Meanwhile, the thermal conductivity (Figure S22c) and tensile strength (Figure S22d) have recovered to 99% (267.8 to 267.1 mW·m^−1^·K^−1^) and 91% (1.68 to 1.54 MPa) of re‐PVBS‐TMC_32_‐D and 99% (267.8 to 266.3 mW·m^−1^·K^−1^) and 86% (1.68 to 1.45 MPa) of re‐PVBS‐TMC_32_‐H respectively.

### 2.4 Flexible Design of Double‐Brush PCPNs and Their Functional Composite

**FIGURE 5:**
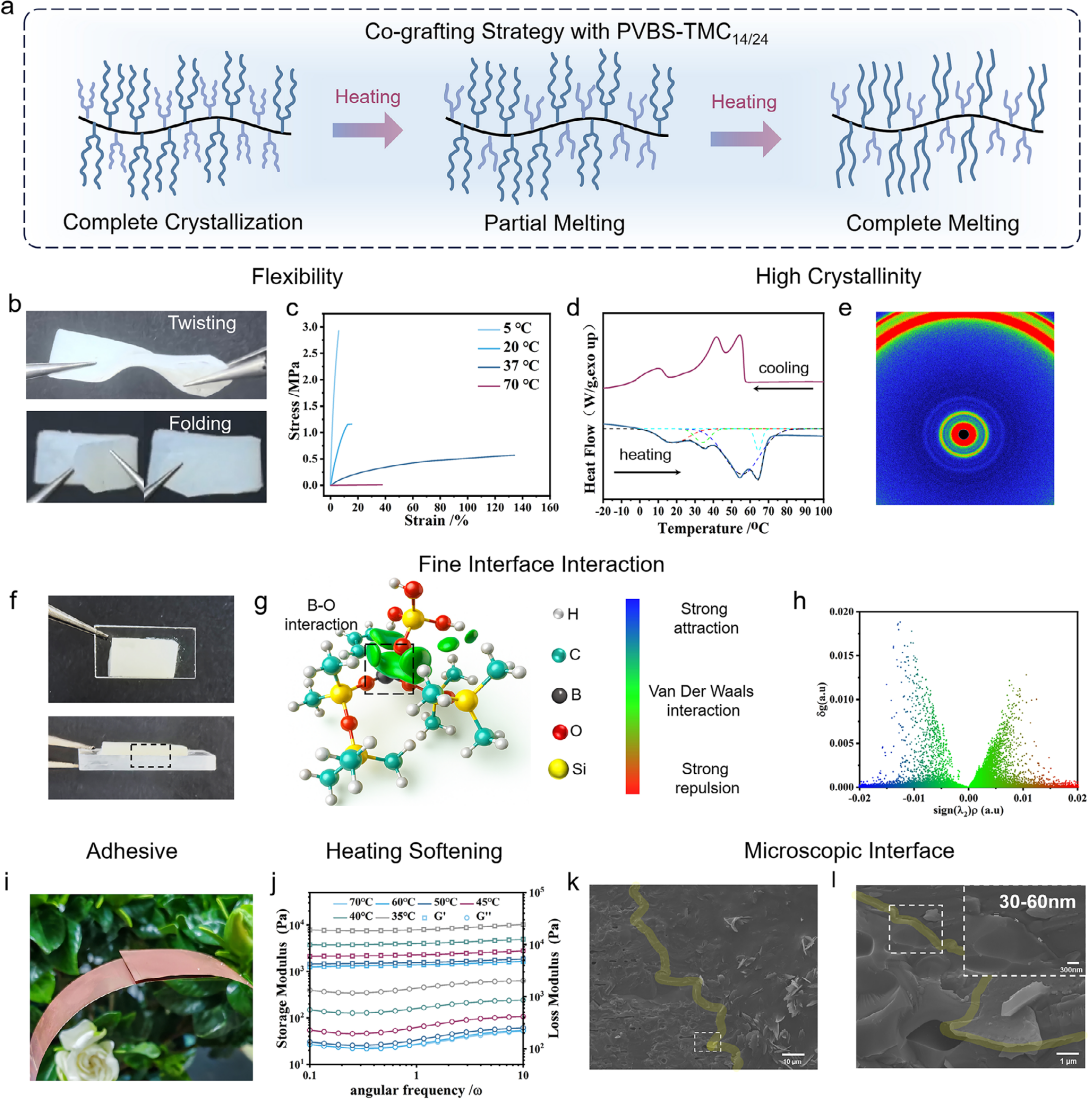
Flexible double‐brush PCPNs based on co‐grafting strategy and their contact, bonding and infiltration behavior. (a) Preparation of flexible PVBS‐TMC_14/24_ by co‐grafting strategy. (b) Free torsion and bending at room temperature of PVBS‐TMC_14/24_ and their (c) stress–strain curves and (d) DSC curves. (e) The SAXS results of PVMS‐TMC_14/24_(red: high intensity, blue: low intensity) (f) Interfacial model of PVBS‐TMC_14/24_ with quartz. (g) IGMH simulation calculation of B─O bonds. (h) Scatter maps between δg and sign (𝜆_2_) 𝜌 of PVBS segment and OH. (i) Adhesion visual image, (j) variable temperature rheological test curve of PVBS‐TMC_14/24_. (k‐l) SEM image of the interface of the interfacial model.

To meet the flexible interface bonding required by electronic devices, we designed flexible PCPNs based on the long and short chain co‐grafting strategy [40]. PVBS‐TMC_12_ and PVBS‐TMC_14_ can be utilized as flexible matrix, but their Δ*H* are limited to 65.4 and 103.9 J·g^−1^, respectively. Longer alkyl chains (such as TMC_28_, TMC_30_, TMC_32_) lead to complete crystallization at room temperature, resulting in loss of flexibility. By co‐grafting TMC_14_ and TMC_24_ in equimolar ratios onto the PVBS backbone (Figure 5a), high‐enthalpy flexible PBS‐TMC_14/24_ was developed. This material remains flexible at body temperature (37°C) (Figure S23), as demonstrated by its ability to twist, fold, and compress without fracture (Figure 5b). In addition, commercial dyes were incorporated in PVBS‐TMC_14/24_ to color the material into red, green, blue, and other hues (Figure S24), offering potential aesthetics applications in smart equipment.

DMA was conducted to evaluate the mechanical properties of PVBS‐TMC_14/24_ at varying temperatures (Figure 5c). At 5°C, all alkyl chains in network are frozen, leaving only the PVBS backbone and amorphous regions mobile, resulting in high tensile strength (3.0 MPa) but significant brittleness (5.9% elongation). At 20°C, partial melting of shorter chains, as evidenced by the first endothermic peak in the DSC curve (Figure 5d), increases chain mobility, leading to improved elongation (15.4%) and reduced tensile strength (1.2 MPa). At 37°C, the complete melting of grafting TMC_14_ crystalline domains allows half of the grafted chains to move freely, while the remaining grafting TMC_24_ crystalline regions act as crosslinking points. This architecture mimics thermoplastic elastomers, yielding exceptional elongation (154.7%) and moderate tensile strength (0.6 MPa), highlighting the flexibility of the double‐brush polyborosiloxane structure. At 70°C, the network relies solely on dynamic boroxine crosslinks, reducing tensile strength to 0.01 MPa and increasing elongation to 49.5%. These mechanical properties confirm the suitability of PVBS‐TMC_14/24_ for applications in flexible electronics and wearable devices, at room/body temperature.

The DSC curves of PVBS‐TMC_14/24_ (Figure 5d) reveals Δ*H*_m_ of 140.7 ± 2.3 J·g^−1^ and Δ*H*_c_ of 136.8 ± 2.5 J·g^−1^. Deconvolution of the curve (black dashed line) identifies four individual peaks (red, blue, green, cyan), where the peaks at 18.7°C and 34.0°C correspond to PVBS‐TMC_14_, contributing 32.8% and 17.9% of total Δ*H* respectively, and those at 55.5°C and 64.5°C belong to PVBS‐TMC_24_, 41.1% and 8.2% respectively. This distribution aligns with the equimolar grafting ratio. However, the experimental Δ*H* (140.7 J·g^−1^) is lower than the theoretical value (166.8 J·g^−1^), attributed to disrupted crystallinity in PVBS‐TMC_24_ due to the incorporation of shorter chains. These amorphous regions enhance flexibility while maintaining high Δ*H*.

WAXS/SAXS analysis elucidated the relationship between condensed‐state structure and flexibility (Figure 5e; Figure S25a,b). Both PVBS‐TMC_14/24_ and PVBS‐TMC_24_ exhibit isotropic crystalline structures, evidenced by concentric diffraction rings (red represents high intensity, blue represents low intensity). PVBS‐TMC_24_ displays broad and intense rings indicating high crystallinity while PVBS‐TMC_14/24_ shows characteristics of the amorphous region at high diffraction angles, and a diffuse green ring at lower diffraction angles, signifying decreased long‐range structure orderliness. Bragg analysis and Debye–Scherrer formula (Table S6) indicate partial integration of shorter chains into the long‐chain lattice, disrupting long‐range order while preserving overall crystallinity. These structural features explain the coexistence of high enthalpy and flexibility.

With leveraging the mechanical and thermal properties of PVBS‐TMC_14/24_, a flexible Cu/Ni‐PET fabric composite (Cu/Ni‐PVBS) was fabricated by coating both sides of the fabric with PVBS‐TMC_14/24_ with the total thickness of 0.2 mm (Figure S26a,b). This composite exhibits electrothermal (Figure S27a,b) and magnetothermal (Figure S27c,d) responsiveness, enabling self‐healing under external stimuli. A 40 µm scratch (Figure S28a) was nearly disappeared after applying 5 V for 60 s (Figure S28b). Similarly, Cu/Ni‐PVBS was exposured to a 2200 W electromagnetic field (shielded by aluminum foil) to induce phase transition and then complete the healing process within 5 s, where the temperature was up to 67.8°C.

The flexibility of composite at 37°C allows effortless bending (Figure S26b). The shielding effectiveness (SE) analysis (Figure S29a) shows enhanced reflection (SER, Figure S29b) due to impedance mismatch at the metal‐polymer‐air interfaces and reduced absorption (SEA, Figure S29c) as the insulating phase‐change layer limits electromagnetic wave penetration [55]. Cu/Ni‐PVBS maintains high EMI SE (∼61 dB) compared to Cu/Ni‐PET (∼64 dB) (Figure S29a), with minimal performance degradation. Electromagnetic interference (EMI) shielding studies revealed that stacking six composite layers (thickness  ≈  0.9 mm) blocks wireless charging signals of smart watch (18 W) (Figure S30). Therefore, Cu/Ni‐PET with temperature control and maintenance function can be applied in protective maternity wear, due to their fine regulation and EMI shielding.

### 2.5 Contact, Bonding, and Infiltration Behavior of PVBS‐TMC_14/24_

PVBS‐TMC_14/24_ revealed exceptional adhesive performance to plain paper (Figure S14), which is primarily attributed to supramolecular interactions involving boron atoms at interface and the ultra‐low modules of double‐brush networks. To investigate these, crosslinked PVBS‐TMC_14/24_ specimens were placed on quartz surfaces, heated to 80°C for 5 min, and cooled to form model interfaces (Figure 5f). The interaction between boron atoms and surface hydroxyl oxygen atoms was computationally evaluated using the Independent Gradient Model based on Hirshfeld partition (IGMH). Simulation results (Figure 5g) clearly demonstrate attractive van der Waals forces, providing theoretical support for adhesive behavior. Interaction strength was further quantified via the sign (λ_2_) ρ function mapped onto IGMH isosurfaces (Figure 5h), where color gradients indicate force intensity, green denotes van der Waals interactions, while blue regions signify stronger intermolecular attractions, confirming predominant B─O van der Waals forces with inherent attractive tendencies [56, 57, 58, 59, 60, 61].

Moreover, the adhesive properties were characterized through substrate compatibility tests. Cured disk samples exhibited universal adhesion to diverse surfaces including wood, polytetrafluoroethylene, aluminum, glass, and leather, with measurable mechanical strength demonstrated in copper foil bonding tests (0.3 MPa) (Figure 5i; Figure S31a–f). This adhesion mechanism arises not only from the aforementioned B─O interactions but also from mechanical interlocking facilitated by continuous thermal softening and fine surface conformability. In situ rheological characterization (Figure 5j) revealed a one‐order‐of‐magnitude modulus reduction (10^4^ Pa to 10^3^ Pa) during the process from 35°C to 70°C, while full crystallization at lower temperatures drastically increased modulus (*G*’ ≈ 2 MPa). The rapid modulus decline upon melting promotes optimal surface contact, with modulus stabilization at elevated temperatures confirming structural stability and self‐adaptivity mechanical property under heat stimulation. Water contact angle measurements (Figure S32) exhibited anomalous behavior, and the crystalline state displayed hydrophobicity (100°), consistent with hydrophobic siloxane/alkyl segments. However, the molten state rapidly transitioned to hydrophilicity (39°). In situ‐XPS analysis (Figure S33) revealed a higher concentration of electron‐deficient boron atoms at 80°C compared to room temperature. This confirms the surface migration of boron atoms during heating and their hydrophilic nature in the molten state.

To further examine interfacial contact, SEM imaging of the quartz‐PVBS‐TMC_14/24_ interface revealed conformal adhesion across multiple magnifications (Figure 5k,l). Yellow dashed lines demarcate the interface between quartz and PCPNs. The measured interfacial width of 30–60 nm confirms exceptional conformal contact, further supporting the foundation for improved interfacial contact and application potential in TIMs.

### 2.6 Self‐Adaptive ITR and Thermal Management of PVBS‐TMC/GF

**FIGURE 6:**
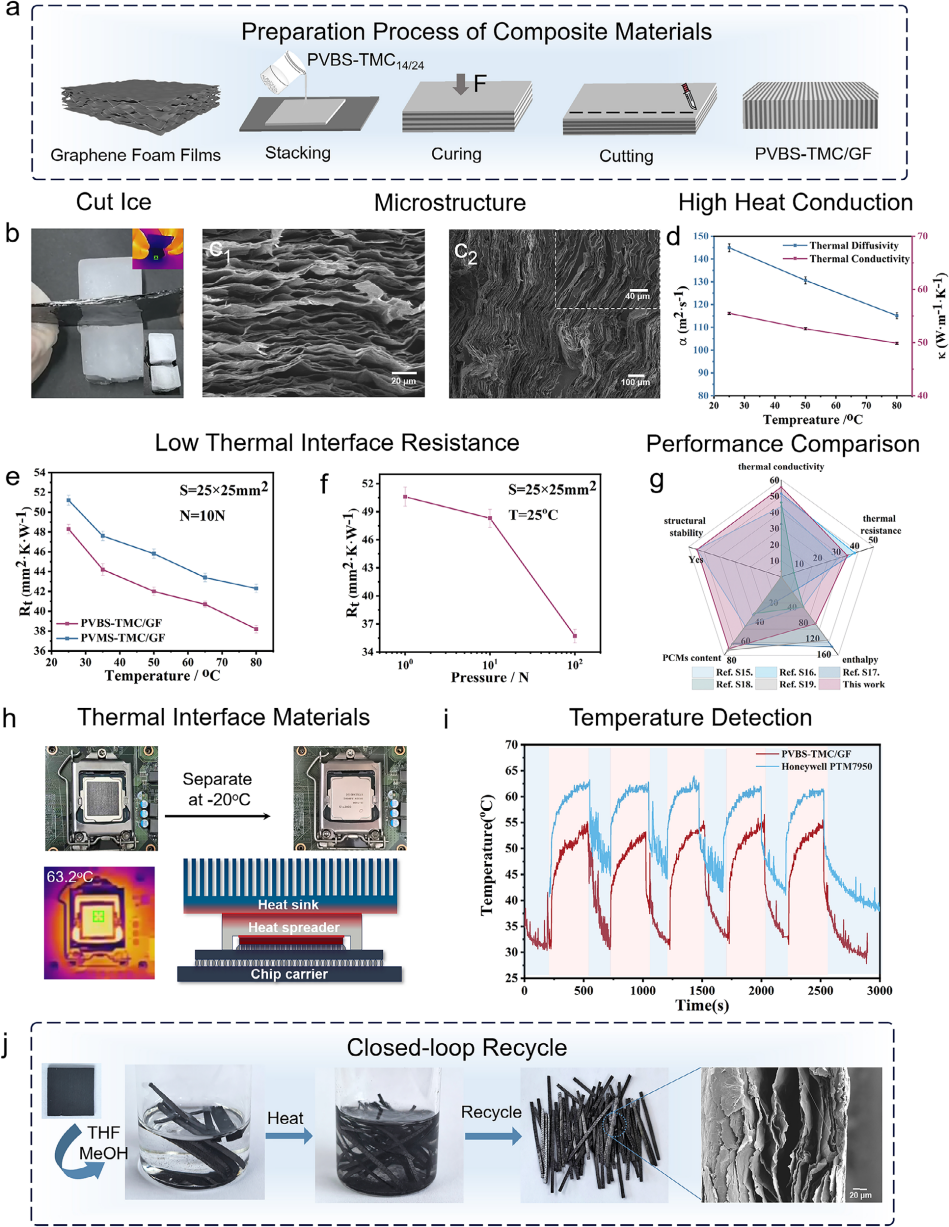
Preparation and application of TIMs based on PVBS‐TMC/CF. (a) The preparation process of PVBS‐TMC/CF. (b) Schematic diagram of ice cutting for single‐layer GF. (c_1_) SEM image of GF without PVBS‐TMC_14/24_ filled. (c_2_) SEM image of PVBS‐TMC/CF. (d) Thermal conductivity k and thermal conductivity α, (e) relationship between temperature and ITR, (f) relationship between pressure and ITR of PVBS‐TMC/CF, and (g) comparison with other reported PCM‐TIMs. (h) Visual image of detachable behavior, infrared imaging, and schematic diagram of the PVBS‐TMC/CF. (i) Temperature curves of CPU equipping PVBS‐TMC/CF and Honeywell PTM7950 within five heating‐cooling cycles. (j) The closed‐loop recycle process of PVBS‐TMC/CF.

Benefiting from the inherent flexibility and unique interfacial characteristics of PVBS‐TMC_14/24_, this material demonstrates broad applicability in TIMs and device thermal management. However, the intrinsically low thermal conductivity of PVBS‐TMC_14/24_ necessitates incorporating thermally conductive fillers, which compromises flexibility. Graphene foam films, a layered graphene‐based carbon material, features micron‐scale lamellar structures and exhibits exceptional thermal conductivity and flexibility due to its abundant π‐electron system and porous architecture [62]. Here, the stacking‐cutting strategy was developed to fabricate PVBS‐TMC/GF composites. As illustrated in Figure 6a, uncured PVBS‐TMC_14/24_ and GF sheets were alternately stacked. To address the optical opacity of GF, 2,5‐Dimethyl‐2,5‐di(tert‐butylperoxy)hexane (DBPH) instead of 1173 employed as radical initiator. Notably, a combined high‐temperature and vacuum‐assisted curing process ensured uniform infiltration of the PVBS‐TMC_14/24_ into the micropores of GF. After curing and diamond wire cutting, the resulting composite exhibited multiple phase‐change peaks between 10°C and 75°C, whereby the phase‐change peak at lower temperatures ensured material flexibility exhibiting Δ*H*_m_ of 99.7 J·g^−1^ (Figure S34).

Figure 6b displays a photograph and infrared image of a single‐layer PVBS‐TMC/GF composite cutting through a 2 cm‐thick ice block at room temperature within 3 min, with heat supplied solely by hand and ambient air, owing to the high thermal conductor of GF (Figure 6c, _1_). Figure 6c_2_ shows the internal morphology of composite, with an inset revealing that PVBS‐TMC_14/24_ infiltrate into GF interstices. Higher‐magnification SEM images (Figure S35a,b) confirm seamless integration without discernible interfaces, providing microstructural evidence for the thermal conductivity of composite. Benefit from the thermal conductor of GF, composite was measured a maximum out‐of‐plane thermal conductivity of 55.5 W·m^−1^·K^−1^ and thermal diffusivity of 114.9 mm^2^·s^−1^ (Figure 6d) by laser flash analysis. In contrast, the in‐plane thermal conductivity of this composite material is significantly reduced to 0.4 W·m^−1^·K^−1^ compared to the out‐of‐plane thermal conductivity. This is due to the disconnection of the heat conduction path in the in‐plane direction of GF.

ITR measurements conducted according to ASTM‐D5470 standards (Figure 6e) revealed a room‐temperature value of 48.3 K·mm^2^·W^−1^ at 10 N (3 psi), which decreased rapidly with increasing temperature. This reduction arises not only from enhanced electron‐phonon transport of GF at elevated temperatures but also from multistage phase transitions of PVBS‐TMC_14/24_ (Figure 5d). Meanwhile, the modulus continuous responds under thermal stimulation (decreasing 10^6^ Pa at 30°C to 10^3 ^Pa at 70°C) and dynamic network improves conformal contact with the heat sink and substrate. This self‐adaptive ITR under increasing temperature enable better interfacial contact and thermal management effectiveness at high temperatures. The result achieved an ultra‐low ITR of 38.2 K·mm^2^·W^−1^ at 80°C upon full melting, surpassing non‐dynamic networks (PVMS‐TMC/GF), composing with PVMS‐TMC_14/24_ and GF, and conventional solid‐solid PCM‐TIMs (Figure 6g; Table S7). Moreover, pressure‐dependent studies demonstrated decreasing thermal resistance from 50.6 K·mm^2^·W^−1^ (1 N, 0.23 psi) to 35.7 K·mm^2^·W^−1^ (100 N, 25 psi) (Figure 6f), attributed to the vertically aligned GF enabling deformation under pressure, thereby ensuring optimal interface contact of TIMs.

In contrast, PVBS‐TMC_32_/GF demonstrates high Δ*H*, however, its ITR is markedly increased  (Figure S36a). This is because PVBS‐TMC_32_/GF remains in a highly crystalline state until its melting point, making it difficult for this rigid composite material to maintain conformal contact with the interface. This lack of contact caused by rigidity leads to geometric mismatch, further producing led to severe phonon scattering as explained by the Kapitza resistance model [7], thereby rendering PVBS‐TMC_32_/GF unsuitable for thermal interface materials. Consequently, we designed the flexible PVMS‐TMC_14/24_ to reduce interfacial thermal resistance through flexible matrix to reduce geometric mismatch and the introduction of dynamic bonds to enhance interfacial coupling and phonon transmission. Meanwhile, the thermal conductivity of PVBS‐TMC_32_/GF composite material is almost determined by the GF, and there is no significant difference from the flexible material, proving the effectiveness of stacking‐cutting strategy (Figure S36b).

Practical validation of PVBS‐TMC/GF was conducted in CPU thermal management (Figure 6h). The composite was applied between a chip and heat sink as TIMs, aligning the GF layers parallel to the heat flux direction (Figure S37). The low ITR and flexibility of composite ensured conformal contact, eliminating air gaps. Figure 6i depicts a schematic illustration and photographic image of the TIM, alongside thermal infrared images under high‐temperature conditions (Figure S38). Under full load (multiple PowerShell processes running for 5 min, repeated over five cycles), PVBS‐TMC/GF reduces CPU steady‐state temperatures by 10°C–15°C and peak temperatures by 8°C–10°C compared to commercial PCM‐TIMs (Honeywell PTM7950) (Figure 6i). It is worth noting that when temperature is below the *T*_m_ of PVBS‐TMC_14/24_, crystallization restricts molecular chain movement and thereby reduces adhesion. Consequently, the mechanical interface bonding force weakens, allowing the TIMs to be readily removed from the CPU. Significantly, no leakage or delamination residues were observed on the surface (Figure 6h). Therefore, replaceable PVBS‐TMC/GF is benefit for disassemble of terminal unit. In addition, we also tested PVMS‐TMC/GF without dynamic bonds by temperature detection (Figure S39), resulting that the temperature of CPU is between PVBS‐TMC/CF and Honeywell PTM7950 under peak and steady state, proving effectivity of dynamic networks. Therefore, PVBS‐TMC/CF with effective thermal management ability highlights the efficiency of the layered structure, where GF rapidly conduct heat to the heat sink, while the phase change ability mitigates thermal peaks and dynamic networks leads the self‐adaptive ITR to assist conduction of heat quantity in high temperature.

Moreover, composites can be extracted into GF through the combined action of THF and MeOH (Figure 6j; Figure S40). The interface of GF remained intact, allowing for the recovery of high‐value GF. Furthermore, PVMS‐TMC_14/24_ can also be recovered from the degraded materials. Employing the recycled PVMS‐TMC_14/24_ and GF, non‐directional TIMs were fabricated, enabling the feasibility of closed‐loop recycling for composites.

## 3 Conclusion

This work proposes and validates a strategy, that ultra‐fast dynamic bonds incorporated into PCPNs effectively reduce Δ*H*_loss_ and enhance interface infiltration while simultaneously combining the high theoretical Δ*H* with the ultra‐low modulus of the double‐brush architecture, thereby achieving a record‐high Δ*H* and self‐adaptive low ITR in TIMs. The double‐brush phase‐change polyborosiloxane network delivers high density of phase‐change units and minimal entanglement, while ultra‐fast‐relaxing dynamic bonds mitigate Δ*H*_loss_ caused by crosslinked structures, thereby breaking the enthalpy ceiling of PCPNs, meanwhile with closed‐loop cyclability. Substantially, we utilize PVBS‐TMC_14/24_ and GF to achieve integrated PCM‐TIMs composites unifying reliability, high Δ*H*, and self‐adaptive low ITR through the construction of stacking‐cutting strategy. Wherein, the low modulus inherent to bottlebrush topology and enhanced interfacial contact of dynamic networks drastically reduces ITR, and GF ensure both flexibility and high thermal conductivity. This work not only establishes a new framework for PCPNs with record Δ*H* but also creates a benchmark for thermal management in high‐flux electronic devices of the AI era.

## Funding

This work was supported by the National Natural Science Foundation of China (Grant Number. 52503261, J.H.M.), the China Postdoctoral Science Foundation (Grant Number. 2025M770100, J.H.M.) and the Fundamental Research Funds for the Central Universities (Grant Number. BH202561, J.H.M.).

## Conflicts of Interest

The authors declare no conflicts of interest.

## Supporting information




**Supporting file**: advs73576‐sup‐0001‐SuppMat.docx

## Data Availability

The data that support the findings of this study are available from the corresponding author upon reasonable request.
