# Supplementary material for: Record‐High Latent Heat, Ultra‐Fast Relaxation and Closed‐Loop Recycling Double‐Brush Polymer Networks for Self‐Adaptive Thermal Interface Management
Source: Adv Sci (Weinh). 2025 Dec 23;13(14):e21482. doi: 10.1002/advs.202521482 (PMC12970164; doi:10.1002/advs.202521482)
Supplement: Supplementary file 1 — Supporting file: advs73576‐sup‐0001‐SuppMat.docx [file ADVS-13-e21482-s001.docx]

**Record-High Latent Heat, Ultra-Fast Relaxation and Closed-Loop Recycling Double-Brush Polymer Networks for Self-Adaptive Thermal Interface Management**

Qiguang Liu^[a]^, Yanyun Li^[a]^, Zhenghao Wu^[a]^, Junjie Cheng^[a]^, Chang Jing^[a]^, Jue Cheng^[a]^, Jiahao Ma^[a]^*, Junying Zhang^[a]^*

[a]College of Materials Science and Engineering, Beijing University of Chemical Technology, Beijing 100029, China

*Corresponding author and E-mail address:

Jiahao Ma: [majiahao@mail.buct.edu.cn](mailto:majiahao@mail.buct.edu.cn); Junying Zhang: [zhangjy@mail.buct.edu.cn](mailto:zhangjy@mail.buct.edu.cn)

**Table of Contents:**

[Experimental Section 2](#_Toc215173143)

[Supplementary Notes 5](#_Toc215173144)

[Figures 6](#_Toc215173145)

[Supplementary Tables 46](#_Toc215173146)

[References 55](#_Toc215173147)

Experimental Section

**Materials**

1-decanol, 1-dodecanol, 1-tetradecanol, 1-hexadecanol, 1-octadecanol, 1-eicosanol, 1-docosanol, 1-tetracosanol, 1-octacosanol, 1-triacontanol, TMC, THF, MeOH, 98% H_2_SO_4_, photoinitiator 1173, DBPH and Tetramethylammonium hydroxide (TMAH) were purchased from Shanghai Macklin Biochemical Co.1-dotriacontanol was purchased from Beijing Warwick Chemical Co. Ltd. D4Vi, VPBA and DDT were purchased from Bide Pharmatech Ltd. And reagents were used without further purification. The Cu/Ni-PET fiber cloth was purchased from YIER in Guangdong with a thickness of 0.08 mm. GF was obtained from Changzhou Fuxi Technology Co., Ltd. The thickness and thermal conductivity of GF are 100 mm, and >100 W·m^-1^·K^-1^, respectively, and the density is 0.2-0.5 g·cm^-3^.

**Synthesis of TMC_n_**

Thiomalic acid derivatives TMC_n_ were synthesized via dehydration esterification between thiomalic acid and a series of alkyl alcohols. Products were designated TMC_10_, TMC_12_, TMC_14_, TMC_16_, TMC_18_, TMC_20_, TMC_22_, TMC_24_, TMC_28_, TMC_30_, and TMC_32_ based on alkyl chain length. Using TMC_32_ as an example, 10 g (0.0214 mol) 1-dotriacontanol and 1.6 g (0.0107 mol) thiomalic acid were dissolved in 15 g THF at 90 ^o^C. After complete dissolution, 0.0585 g concentrated H_2_SO_4_ was added, and the mixture reacted for 3 hours. After reaction, hot water and toluene were added for liquid separation to remove residual acid. Repeated washes achieved pH neutrality, and then residual solution was evaporated via rotary evaporation to yield TMC_n_.

**Synthesis of Phenylboronic Acid-Alkane Derivatives (PAD)**

TMC_32_ and VPBA underwent UV-initiated thiol-ene click reaction with photoinitiator 1173 to form PAD. Specifically, 2 g (0.0135 mol) VPBA, 14.1617 g (0.0135 mol) TMC_32_, and 0.08 g 1173 were dissolved in 30 g THF at 90 ^o^C. The mixture was reacted under UV irradiation (365 nm, ~10 mW/cm²) with stirring for 3 hours. Solvent was evaporated at 70 ^o^C via rotary evaporation, preventing boronic acid condensation, to obtain PAD.

**Synthesis of PVMS**

PVMS was synthesized by D4Vi through open-ring reaction. 100 g D4Vi and 0.5 g TMAH was combined in a flask in 90 ^o^C under the protection of N_2_. After the reaction of 30 minutes, 20g water was added in flask maintain 3 hours. Then, heating to 120 ^o^C and keep 1 hours caused siloxane rearrangement to reduce the proportion of ring siloxane. Last, D4Vi was removed by rotary evaporation to obtain PVMS.

**Synthesis of Polyvinylborosiloxane (PVBS)**

PVBS was synthesized by acid-catalyzed dehydration condensation between PAD and PVMS. A mixture of 20 g PVMS (≈ 0.0032 mol, Mₙ = 3600 g/mol by GPC) and 2 g (0.0017 mol) PAD was combined with 0.5 g concentrated HCl (12 mol/L) in a flask. Under vacuum (0.1 MPa) at 110 ^o^C for 5 h, the reaction proceeded. HCl was removed by rotary evaporation at 120 ^o^C and 10 mbar, yielding PVBS, which can be well dissolved in TMC_n_.

**Synthesis of Trithiol Crosslinker (TSH)**

TSH was synthesized via kinetic-controlled thiol-ene reaction between VPBA and excess DDT, followed by vacuum dehydration forming dynamic boroxine crosslinker. A solution of 3 g (0.0203 mol) VPBA, 15 g (0.0725 mol) DDT, and 0.09 g 1173 in 30 g THF underwent UV irradiation (365 nm, ~10 mW/cm²) with stirring for 3 hours. After reaction, solvent and excess dithiol were evaporated under high vacuum (1 mbar) at 120 ^o^C, inducing boronic acid dehydration to form TSH.

**Preparation of PVBS-TMC_n_ PCPNs**

Alkyl-modified PVBS, TMC_n_ side chains, and TSH crosslinker formed a homogeneous phase above melting points. PVBS was blended with TMC_10_, TMC_12_, TMC_14_, TMC_16_, TMC_18_, TMC_20_, TMC_22_, TMC_24_, TMC_28_, TMC_30_, or TMC_32_ above their respective *T*ₘ. TSH and 1173 (0.5 wt% of total mass) were added, maintaining total thiol:vinyl molar ratio = 1:1 and TSH:TMC_n_ thiol molar ratio = 5:95. The mixture was poured into PTFE molds above *T*ₘ and UV-cured (365 nm, ~100 mW/cm², 1 minute) to yield crosslinked PVBS-TMC_n_ networks.

**Degradation and recycling of PVBS-TMC_32_**

PVBS-TMC_32_ was exemplarily manufactured as a prototype for degradation and recycling studies. During the degradation process, PVBS-TMC_32_ was immersed in mixed solvent (mass ratio is THF:MeOH = 95:5) and heated above its melting temperature for 3 hours to ensure complete degradation. Then, the obtained solution was added to a circular PTFE mold and the solvent was evaporated in a vacuum oven at 120 ^o^C for 24 hours to ensure dehydration of dynamic bonds.

The recycling process is carried out under hot-press. PVBS-TMC_32_ is firstly solidified in a square PTFE mold. The obtained material is then cut into small squares and placed in the hot press. Under a temperature of 100 ^o^C and a pressure of 5 MPa, it is hot-pressed for 1 minute to obtain the reprocessed product.

**Preparation of PVMS-TMC_32_ PCPNs**

Dissolve PVMS and TMC_32_ in THF at a vinyl to thiol molar ratio of 10:1, adding 0.5% 1173, and expose to ultraviolet light for 5 seconds above *T*ₘ. Remove the solvent and set aside for later use. Compatibilized above partial grafting PVMS backbone, TMC_n_ side chains, and TSH crosslinker formed a homogeneous phase above melting points. PVMS was blended with TMC_32_ above their respective *T*ₘ. DDT and 1173 (0.5 wt% of total mass) were added, maintaining total thiol:vinyl molar ratio = 1:1 and DDT:TMC_n_ thiol molar ratio = 5:95. The mixture was poured into PTFE molds above *T*ₘ and UV-cured (365 nm, ~100 mW/cm², 1 minute) to yield crosslinked PVMS-TMC_n_ networks.

**Preparation of PVBS-TMC_14/24_**

PVBS was blended with equimolar TMC_14_ and TMC_24_ above *T*ₘ. TSH and 1173 (0.5 wt%) were added (total thiol:vinyl = 1:1; TSH:TMCn thiol molar ratio = 5:95; TMC_14_:TMC_24_ = 1:1 mol/mol). UV curing (365 nm, ~100 mW/cm², 1 minute) in PTFE molds above Tₘ yielded flexible PVBS-TMC_14/24_.

**Preparation of** **Cu/Ni-PVBS**

Cu/Ni-PVBS was manufactured by Cu/Ni-PET fiber cloth and PVBS-TMC_14/24_. Cu/Ni-PET was placed on a glass substrate and then blade coated with PVBS-TMC_14/24_ precursor. Then, precursor was cured by UV (365 nm, ~100 mW/cm², 1 minute) above *T*ₘ, obtaining Cu/Ni-PVBS containing the single-side layer PVBS-TMC_14/24_. Finally, the same procedure was repeated on the opposite side to get Cu/Ni-PVBS. Total thickness of Cu/Ni-PVBS was maintained 0.2 mm.

**Independent Gradient Models Based on Hirshfeld Partition (IGMH) Simulation**

Combine Molclus 1.12 and xtb-6.5.1 search for the minimum energy conformation of PVBS segments and hydroxyl groups as model, which were optimized at wB97XD/6-311++G (d, p) level with the Gaussian 16 suite of programs. After optimization, the electronegativity of each model was calculated to assess their potential for interaction force by the Multiwfn 3.8 program. IGMH analysis can exhibit interaction force in real space by combining with electron density (𝜌) and the sign of second eigenvalues of the electrondensity Hessian matrix (sign(𝜆_2_)). The color-mapped isosurface graphs of IGMH were rendered by the VMD 1.9.3 program.

**Preparation of PVBS-TMC/GF**

PVBS-TMC_14/24_ precursor was supplemented with 0.5 wt% DBPH. GF sheets (2.5 × 2.5 cm) were alternately stacked with uncured PVBS-TMC_14/24_ in a mold (>25 mm height). The GF was placed on a glass substrate and then blade coated with the pre-mixture with a calculated mass. Second GF was stacked above and followed with blade coating of pre-mixture. Such process was repeated until the height of the stacking bulk reached 25 mm. Under 500 g pressure and vacuum (-25 bar) at 80 ^o^C for 6 h, enabling precursor completely infiltrate into GF. Then it was cured at 135 ^o^C for 2 h to get final PVBS-TMC/GF blocks. PVBS-TMC/GF samples were perpendicularly cut from the block with a diamond wire-cutting machine. At last, the adherent residual PVBS-TMC_14/24_ on the surface of PVBS-TMC/GF was removed by THF/MeOH.

Otherwise, the precursor of PVBS-TMC_32_/CF and PVMS-TMC_32_/CF were PVBS-TMC_32_ and PVMS-TMC_32_ respectively. The preparation methods of these two composite materials are consistent with the above.

**Measurements**

The FT-IR Spectroscopy analysis was performed using a Nicolet Nexus 670 spectrometer in transmission mode over 4000-400 cm^−1^ region with a resolution of 4 cm^−1^ using a KBr crystal plate. DSC measurements were carried out by a TA Q20. All the specimens had their thermal history eliminated and were tested in the temperature range from -50 ^o^C to 120 ^o^C at a heating rate of 10 ^o^C /min. The accuracy of temperature and enthalpy for this instrument is ±0.05 ^o^C and ±0.1%, respectively, while the measurement uncertainty of temperature and enthalpy is ±0.3 ^o^C and ±1.5%, respectively. X-ray diffraction (XRD) measurements were carried out using a Rigaku D/Max 2500V B2+/PC diffract meter with Cu Kɑ radiation over a 2𝜃 range of 5^o^-90^o^. Wide-angle X-ray scattering measurements (WAXS) were carried out on a Xeuss 3.0 (Xenocs SAS, France) SAXS apparatus with Cu Kα X-ray radiation. Rheological analysis was carried out on a stress-controlled Anton Paar MCR 102 rheometer with 25mm parallel plate tools at a force of 1 N. Frequency sweeps were performed from 10^2^ to 10^−1^ Hz at 1% stain at 90 ^o^C. Stress relaxation measurements were conducted in stress - strain mode using the Anton Paar MCR 102 rheometer. Thermal mechanical properties were tested using a Dynamic Mechanical Analyzer (DMA, TA Q800), and the specimen (30mm×6mm×1mm) was examined at a constant frequency of 1 Hz over the temperature ranging from -120 ^o^C to 90 ^o^C with a heating rate of 1 ^o^C/min. The tensile tests were conducted using DMA using controlled force mode (0.1 N/min). The molecular weight distributions and dispersity (Ð) were assessed by gel permeation chromatography Waters 1500 (GPC, Waters Technologies (Shanghai) Limited) using THF eluent at a flow rate of 1 mL/min with Viscotek 305 TDA liquid chromatography. GPC analysis was performed in THF with polystyrene (PS) standards. The XPS spectra of the samples were analyzed by a Thermo Fisher Scientific Nexsa G2. A HIKVISION camera with an emissivity of 0.97 was employed to obtain the infrared thermal images. The temperature change of samples under heating or electrification was recorded using a paperless recorder with thermocouples (UT325F UNI-T). A diamond wire - cutting machine (HA320, Suzhou Sangong Precision Machinery Co., Ltd.) was used. The thermal diffusivity coefficient was tested by using the German NETZSCH LFA 467 laser thermal conductivity meter and converted to get the thermal conductivity coefficient. Thermal interface resistance was tested by using the Xiangtan Xiangyi Instrument Co., Ltd fully automatic tester for thermal conductivity and thermal resistance DRL-III Tested in accordance with ASTM D5470-2017 standard. The adhesion (lap shear) tests were performed according to GB/T 7124-2008 using a SANS UTM5205XHD universal testing machine. The overlap area of bonded specimens was 12.5 mm × 25 mm. At least three replicates were tested for each sample to determine the average adhesion strength. The shielding effectiveness (SE) analysis were carried out using a network analyzer (Agilent E5071C) with a coaxial transmission line fixture, following the ASTM D4935-18 standard. The frequency range for the measurement was 8.2-12.4GHz.

Supplementary Notes

Calculation of power law exponents between G’’ and ω^n^: We fitted the loss modulus and angel frequence data on a log-log plot (G’’ and ω) to the form G’’ ∝ ω^n^ and extracted the slope (n) by linear regression.

Figures


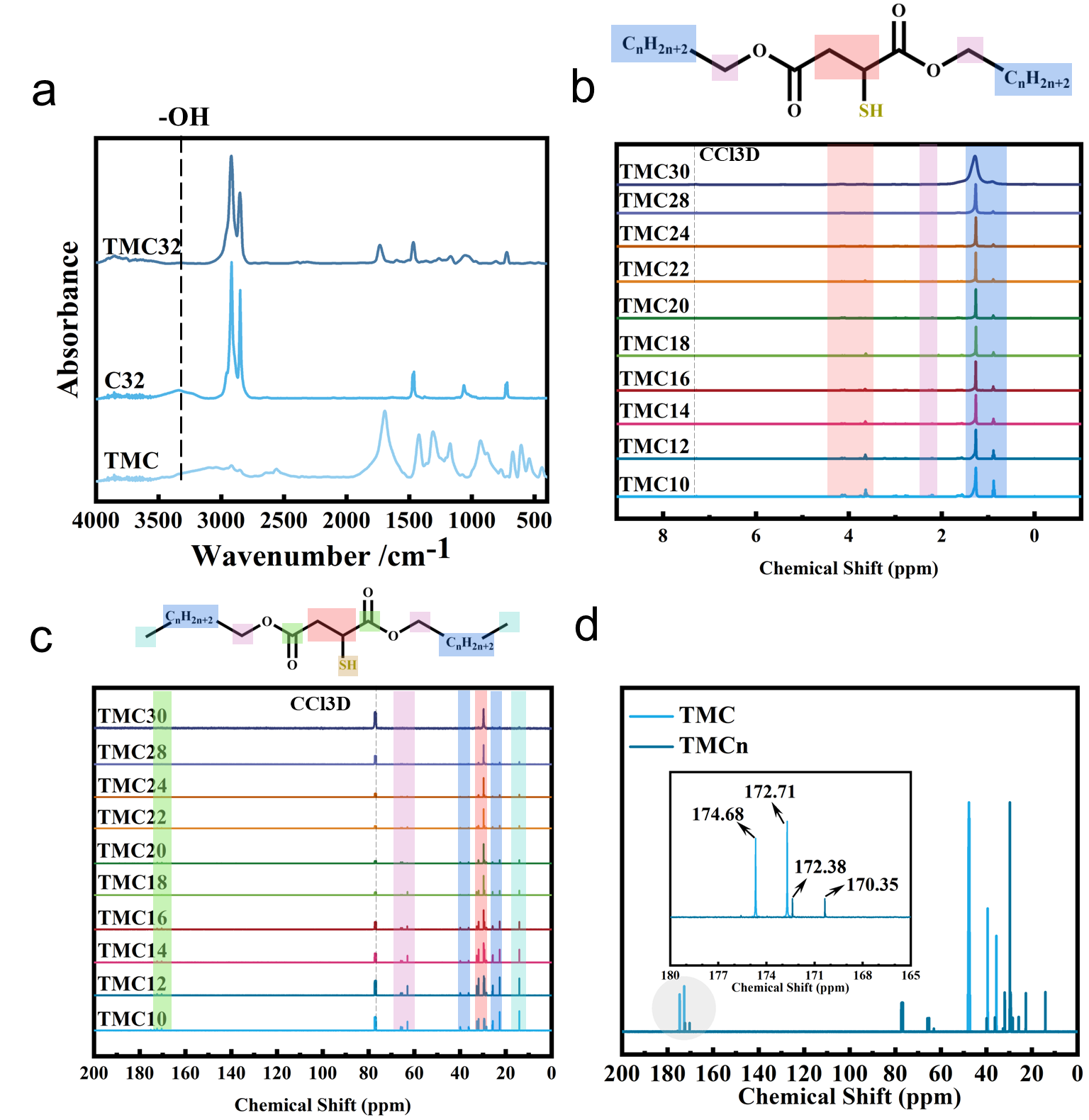


**Figure S1.** (a) FT-IR spectra of TMC_32_, C_32_ and TMC. ^1^H NMR spectra (b) and ^13^C NMR (c) of TMC_n_. (d) The ^12^C NMR spectra of comparison between TMC and TMC_n_.


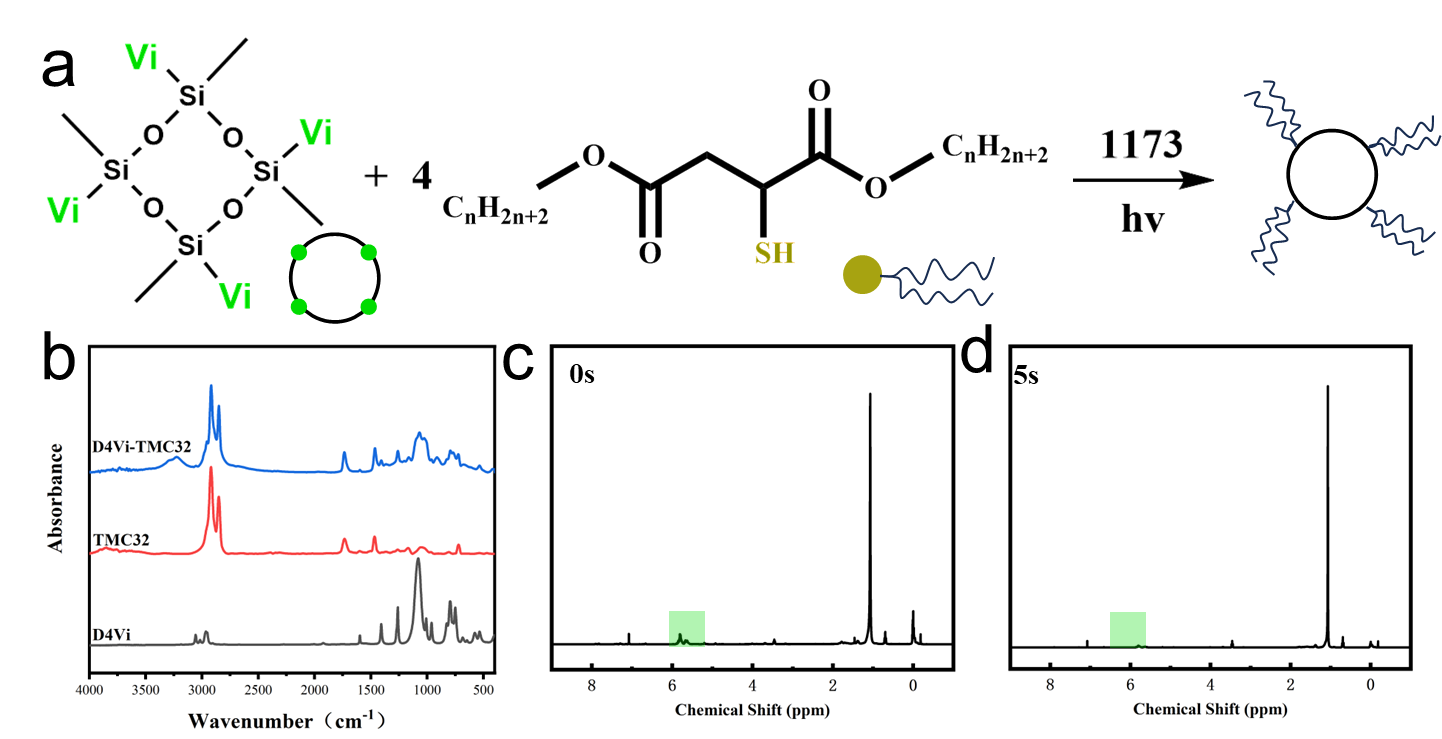


**Figure S2. The thiol reactivity of TMC_n_ was verified by model compounds.** (a) The schematic diagram of click reaction between D4Vi and TMC_n_ by photoinitiator 1173 and UV-light. (b) FT-IR spectra of D4Vi, TMC_n_ and D4Vi-TMC_n_. (c) ^1^H NMR spectra of D_4_Vi, TMC_n_ and photoinitiator 1173 mixture before reacting. (d) ^1^H NMR spectra of mixture after 5 second of UV-light.

Based on the purpose of model exploration to illustrate that the thiol-ene click reaction is fully in progress. D4Vi and TMC_32_ were mixed above their melting point, maintaining a molar ratio of vinyl to thiol at 1:1. Then add 0.5wt%1173 and mix well.

D4Vi and TMC_32_ were subjected to UV irradiation for 5 seconds. The peak in IR at 1598 cm^-1^ of vinyl group nearly disappeared (Figure 2b), and the vinyl proton peaks at 5.8 ppm and 5.6 ppm in the ¹H NMR (Figure 2c, 2d) spectrum were also significantly diminished.


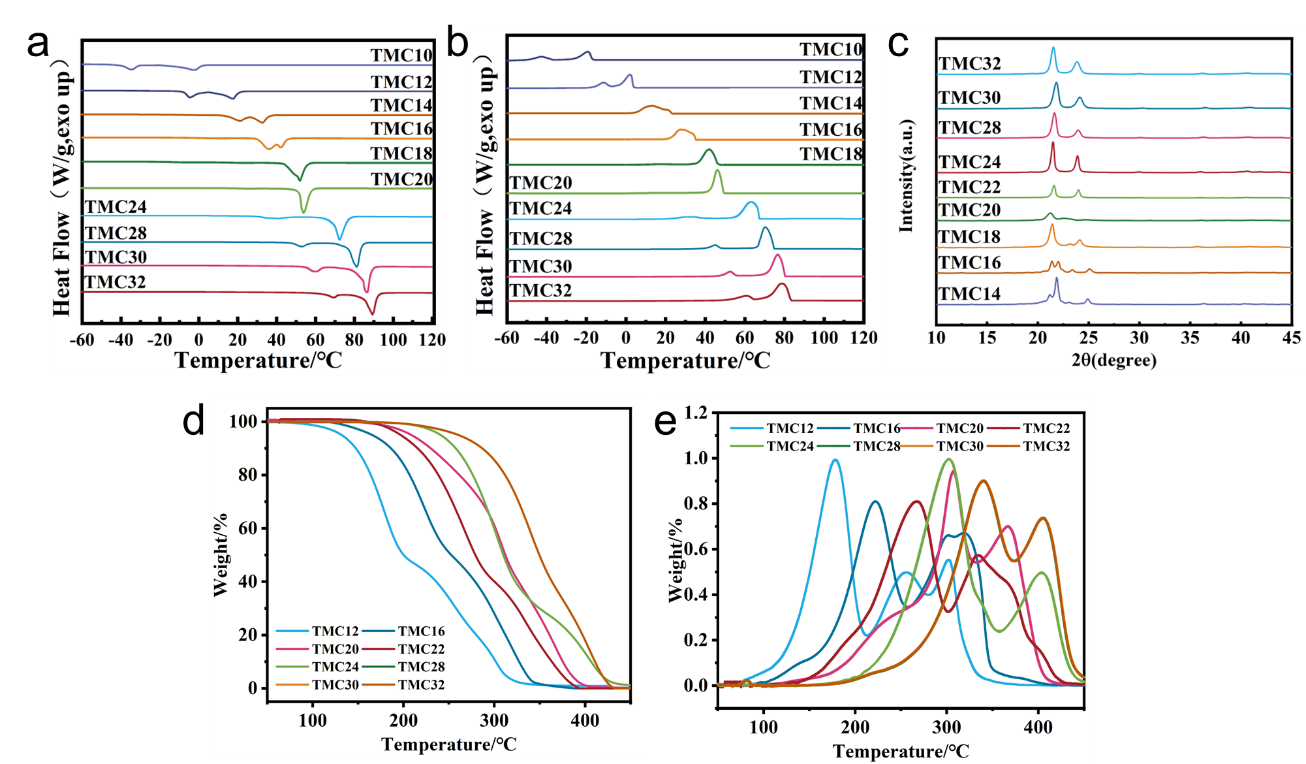


**Figure S3.** The melting process (a) and crystallizing process (b) of DSC, (c) XRD, (d) TGA, and (e) DTG curve of TMC_n_.


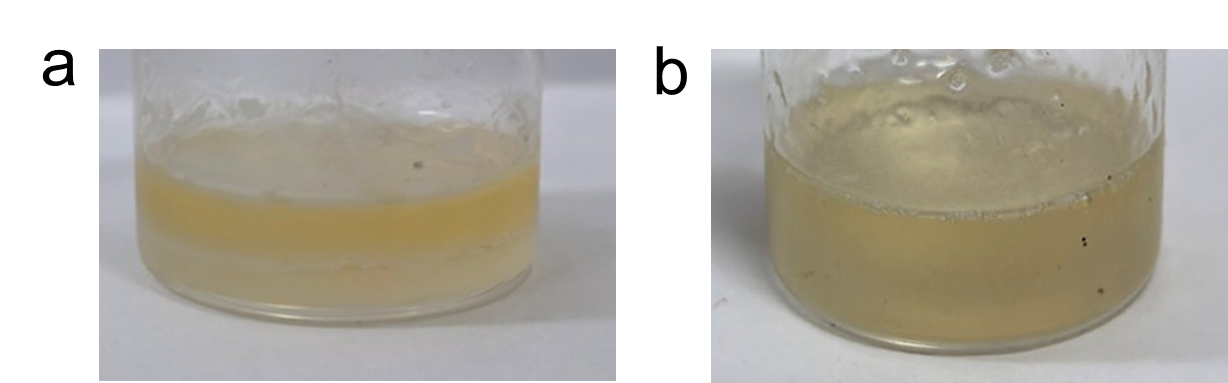


**Figure S4.** (a) Visual photos of PVBS, synthesized by PVMS and VPBA, shows obvious phase separation. (b) Visual photo of PVBS, synthesis by PVMS and PAD, shows obvious homogeneous phase.


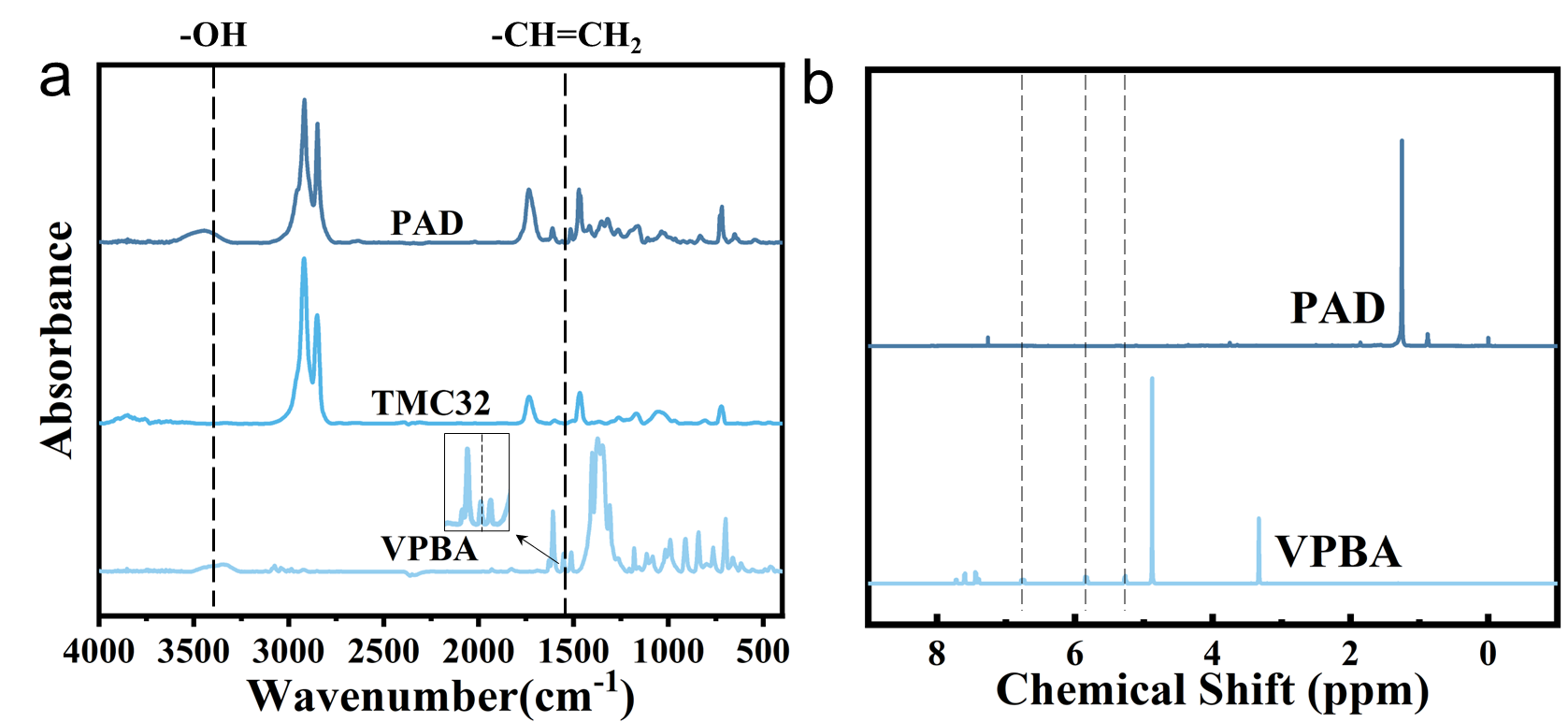


**Figure S5.** (a) FT-IR and (b) ^1^H NMR spectra of PAD, TMC_32_ and VPBA.


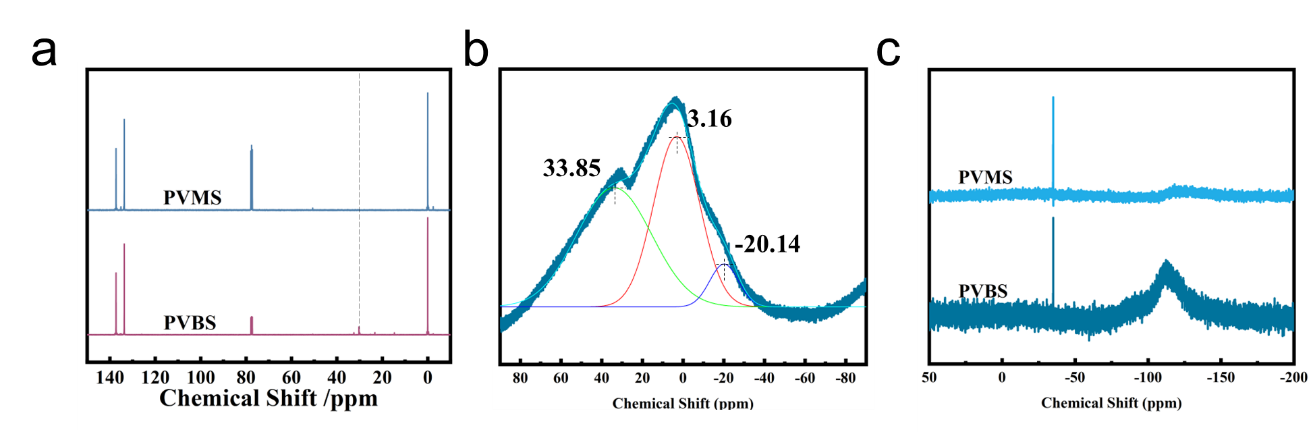


**Figure S6.** (a) ^13^C NMR, (b) ^11^B NMR and (c) ^29^Si NMR spectra of PVMS and PVBS.


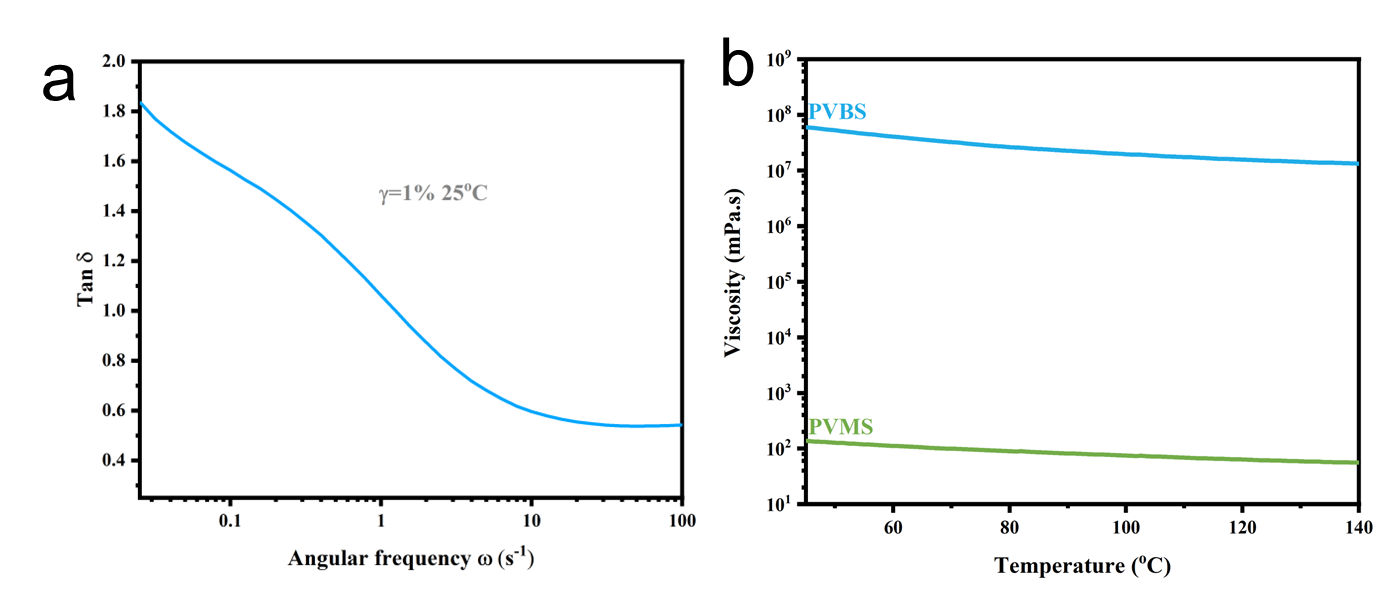


**Figure S7.** (a) Tanδ of PVBS measured by rheometer (shear strain is 1 % and temperature is 25 ^o^C). (b)Viscosity of PVBS and PVMS.


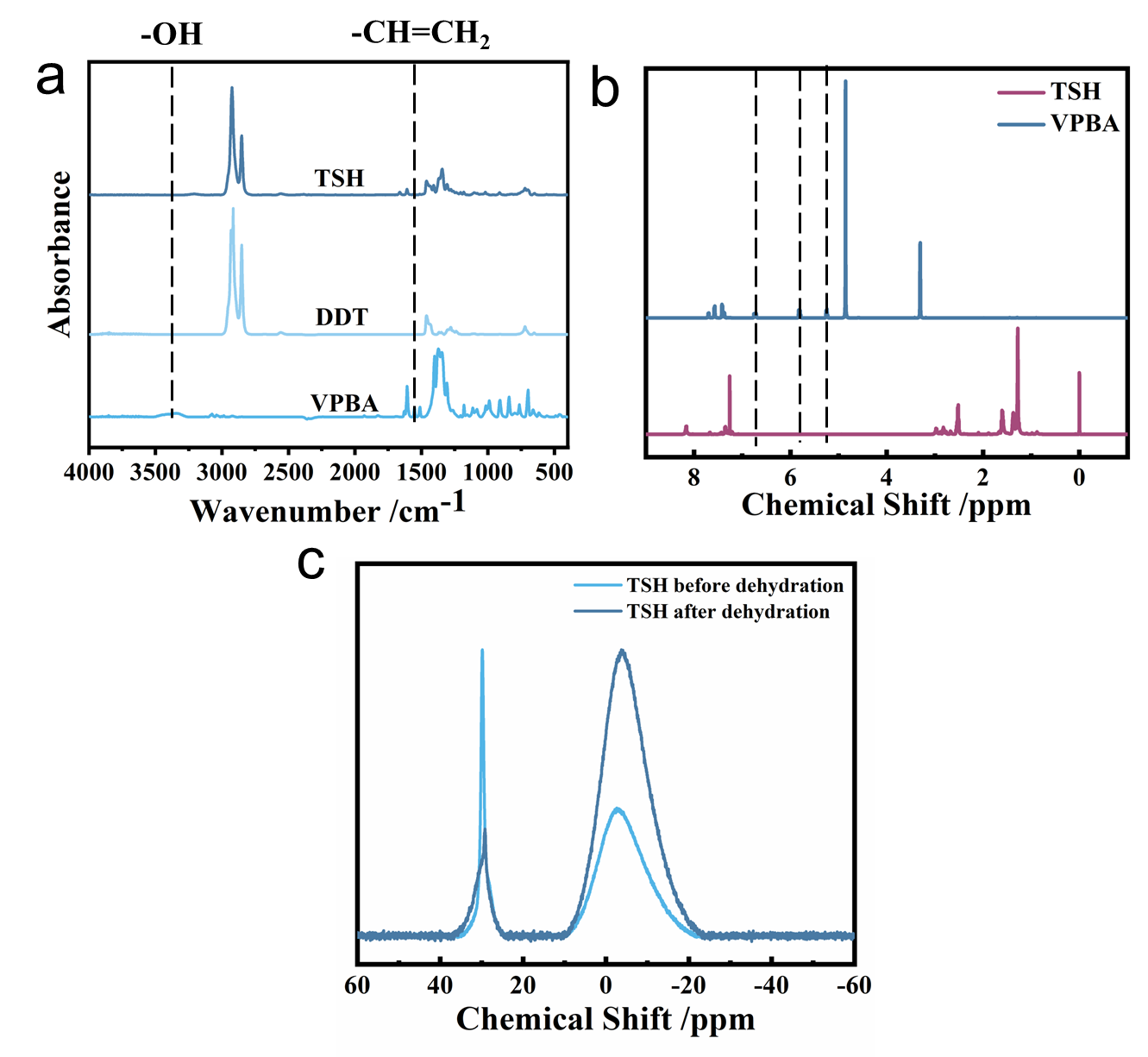


**Figure S8.** (a) FT-IR spectra of TSH, DDT and VPBA. (b) ^1^H NMR spectra of TSH and VPBA. (c) ^11^B NMR spectra of TSH before and after dehydration.


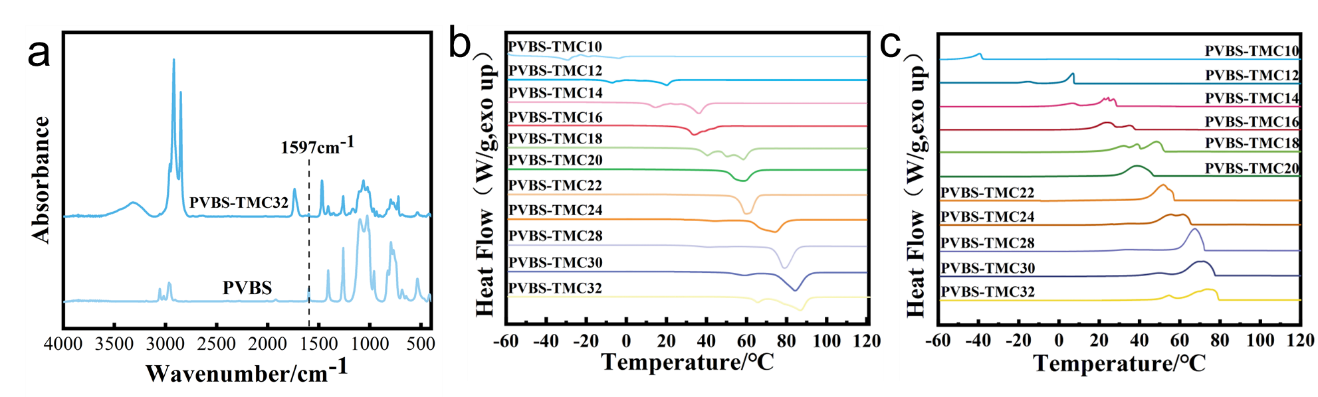


**Figure S9.** (a) FT-IR spectra of PVBS-TMC_32_ and PVBS. (b) The melting process (b) and crystallizing process (c) of PVBS-TMC_n_ in DSC curves.


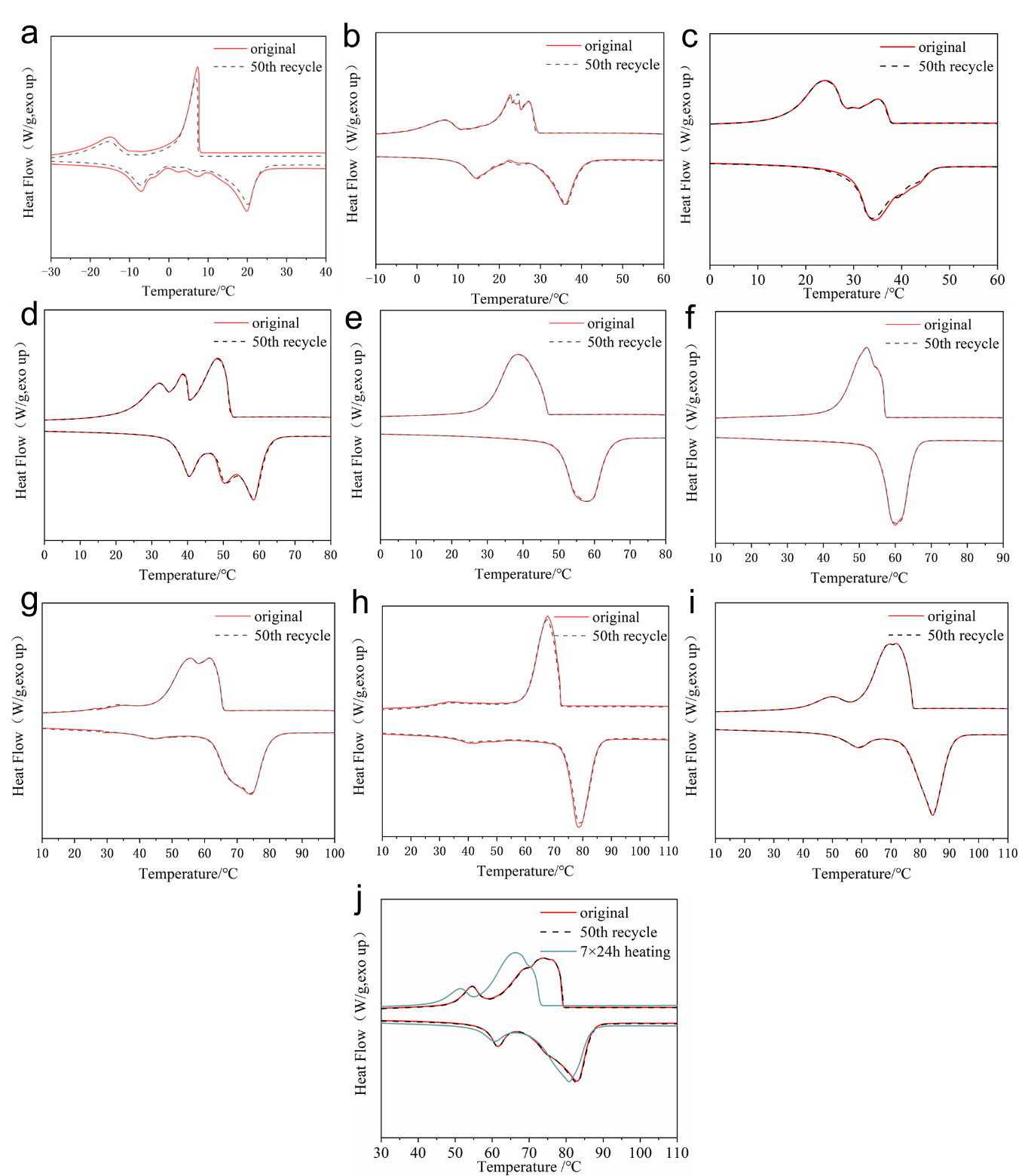


**Figure S10.** Thermal cycling DSC curves of PVBS-TMC_n_ (n = 12(a), 14(b), 16(c), 18(d), 20(e), 22(f), 24(g), 28(h), 30(i),32(j)).


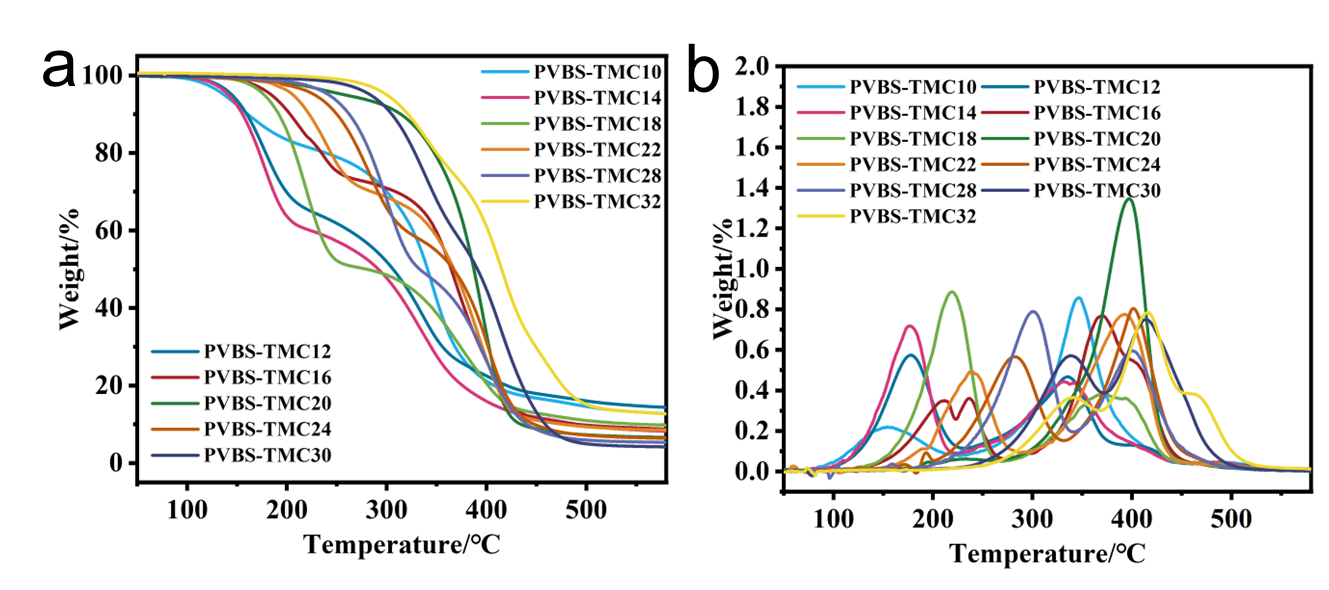


**Figure S11.** TGA (a) and DTG (b) curves of PVBS-TMC_n_.

Two-stage decomposition was observed, lower-temperature degradation (100~300 °C) of alkyl side chains followed by cyclization-driven breakdown of the polyborosiloxane network (350~450 °C). Enhanced char residue in boron-rich networks resulted from disrupted siloxane regularity and residual long-chain inhibition of cyclization.


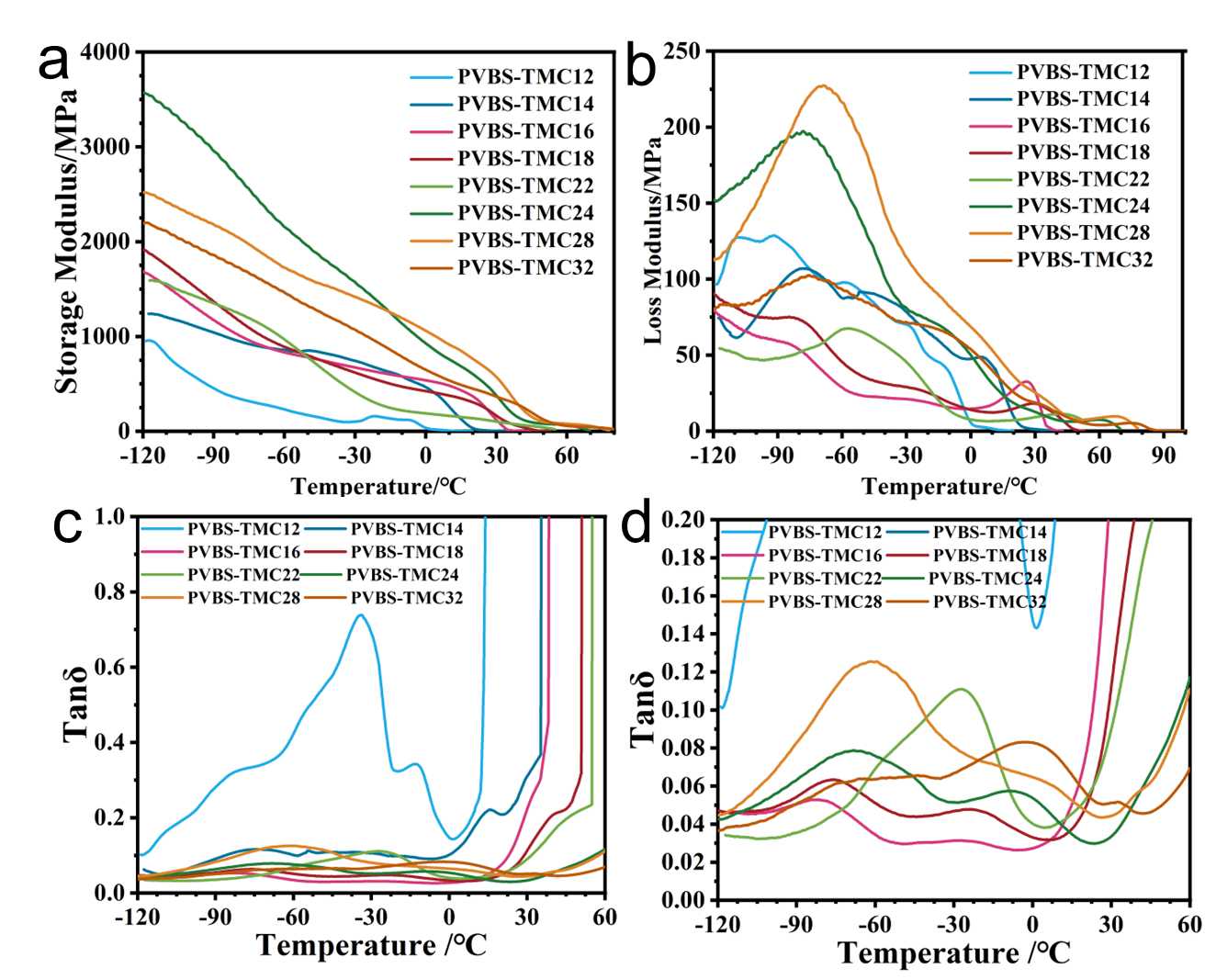


**Figure S12.** The storage modulus (a), loss modulus (b) and Tanδ (c-d) result of PVBS-TMC_n_ measured by DMA.

As Figure 9a, for PVBS-TMC_12_ with shorter chains, polyborosiloxane networks predominantly determined strength, resulting in the lowest modulus. Chain lengths between PVBS-TMC_14_ to PVBS-TMC_22_ enhanced crystallinity, yielding modulus contributions from both crystalline domains and crosslinked networks, thus elevating modulus values. However, PVBS-TMC_24_ and longer chains induced excessive crystallization that disrupted the regularity of the polyborosiloxane network, causing modulus reduction as alkyl crystallites became the dominant contributor.

**
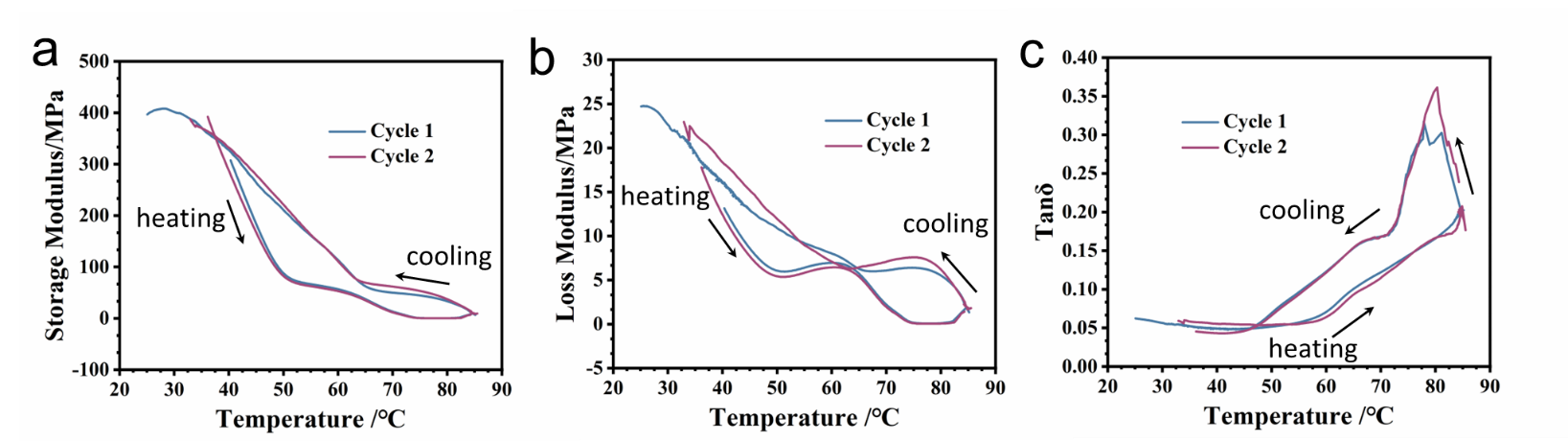
**

**Figure S13.** The cyclic DMA curves of PVBS-TMC_32_.


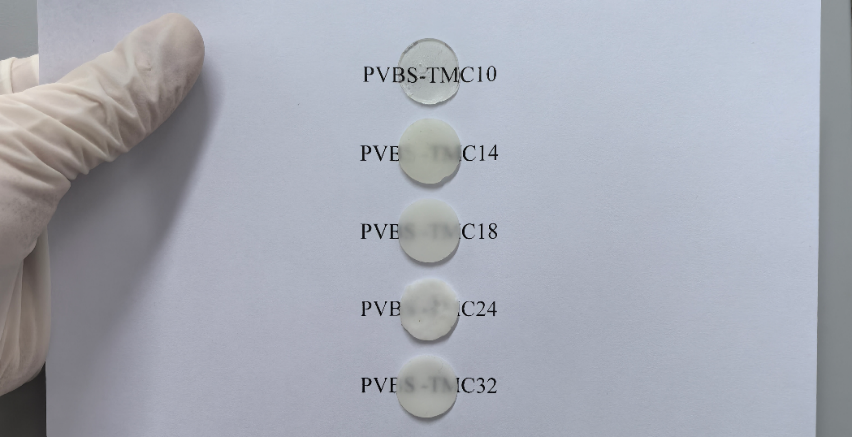


**Figure S14.** The visual image of PVBS-TMC_n_.


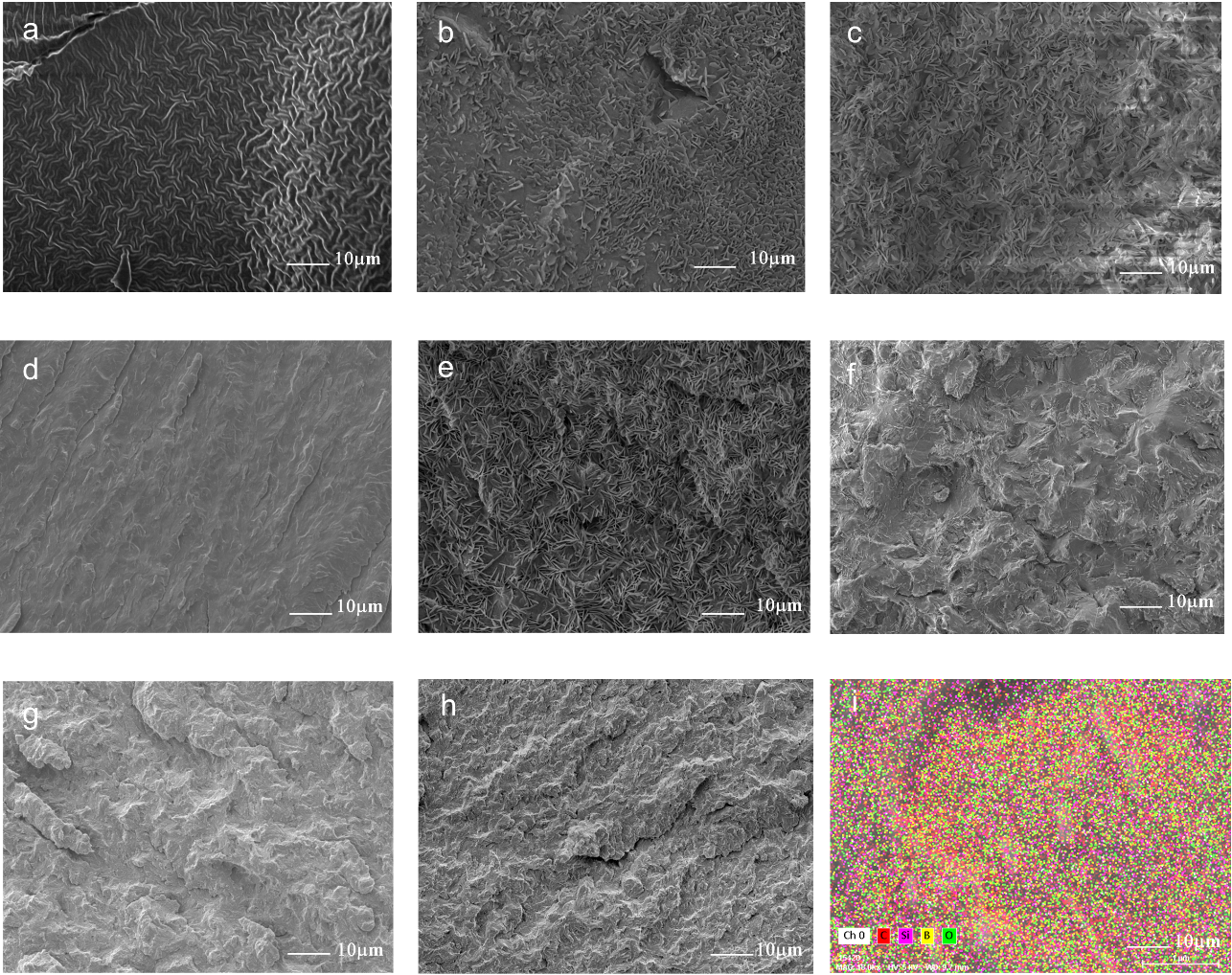


**Figure S15.** SEM image of PVBS-TMC_n_ (n = 10(a), 14(b), 16(c), 20(d), 22(e), 24(f), 28(g) and 32(h)) and SEM-EDS images (i) of PVBS-TMC_n_.

Scanning electron microscopy (SEM) analysis revealed distinct morphological features. PVBS-TMC_10_ exhibited surface wrinkles attributed to electron beam-induced deformation in soft materials, while PVBS-TMC_18_ displayed lamellar crystalline structures along fracture planes due to moderate crystallinity. In contrast, PVBS-TMC_32_ showed brittle fracture surfaces characteristic of highly ordered crystalline domains.


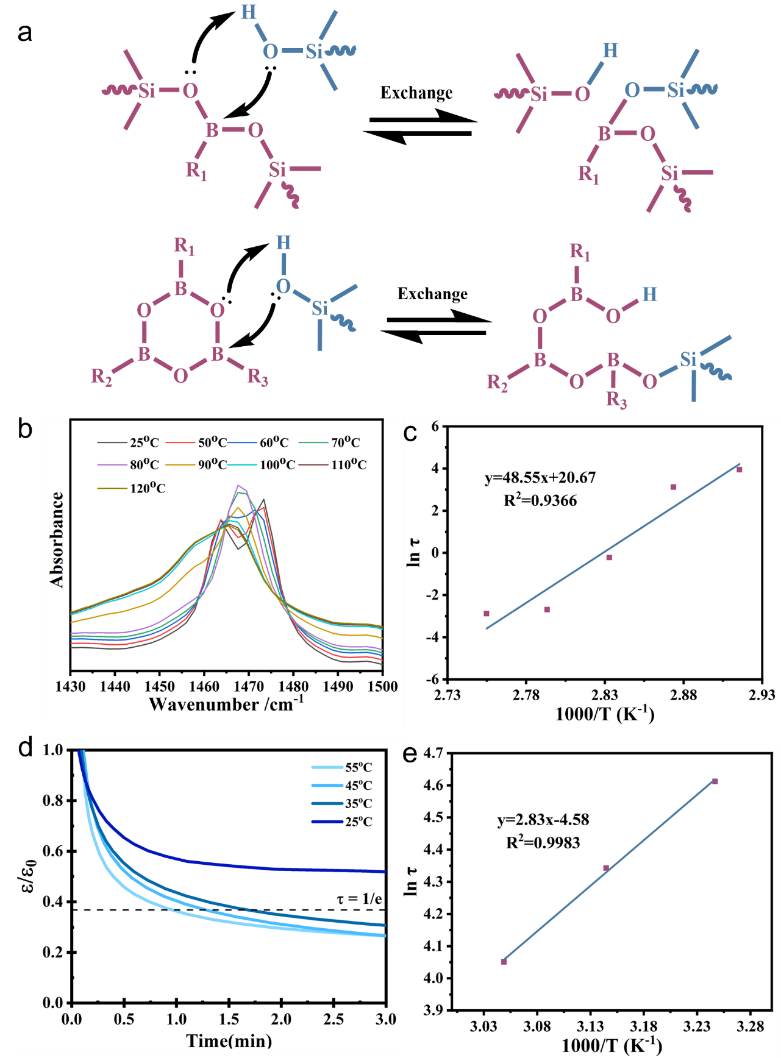


**Figure S16.** (a) The exchange of Si-O-B bond. (b) FT-IR curve of B-O bond in PVBS-TMC_32_ under a series of temperature. (c) Linear fitting of relaxation activation energy of PVBS-TMC_32_. Stress relaxation (d) and linear fitting (e) of model compounds.


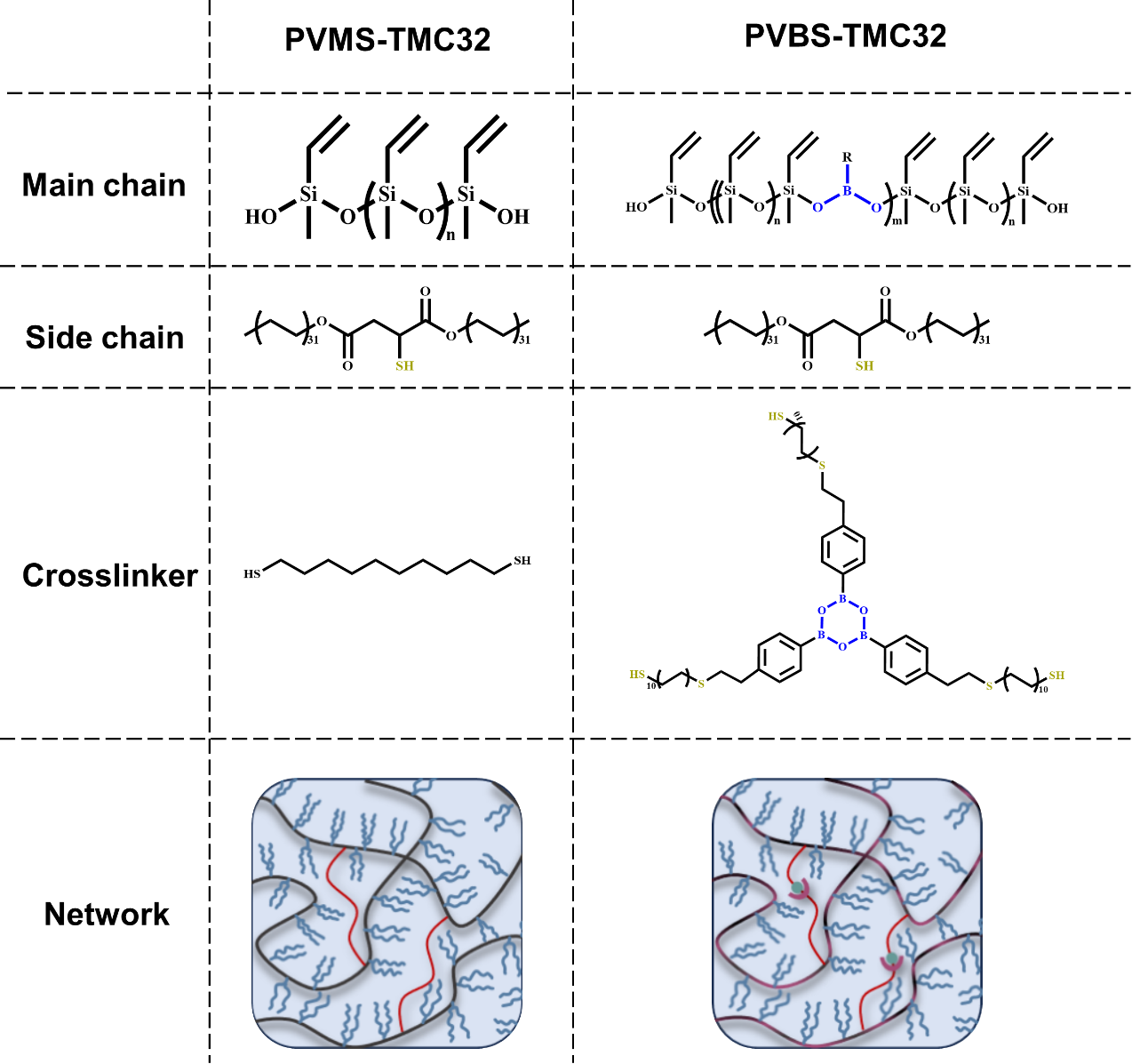


**Figure S17.** The composition of permanent crosslink network (PVMS-TMC_32_) and dynamic crosslink network (PVBS-TMC_32_).

**
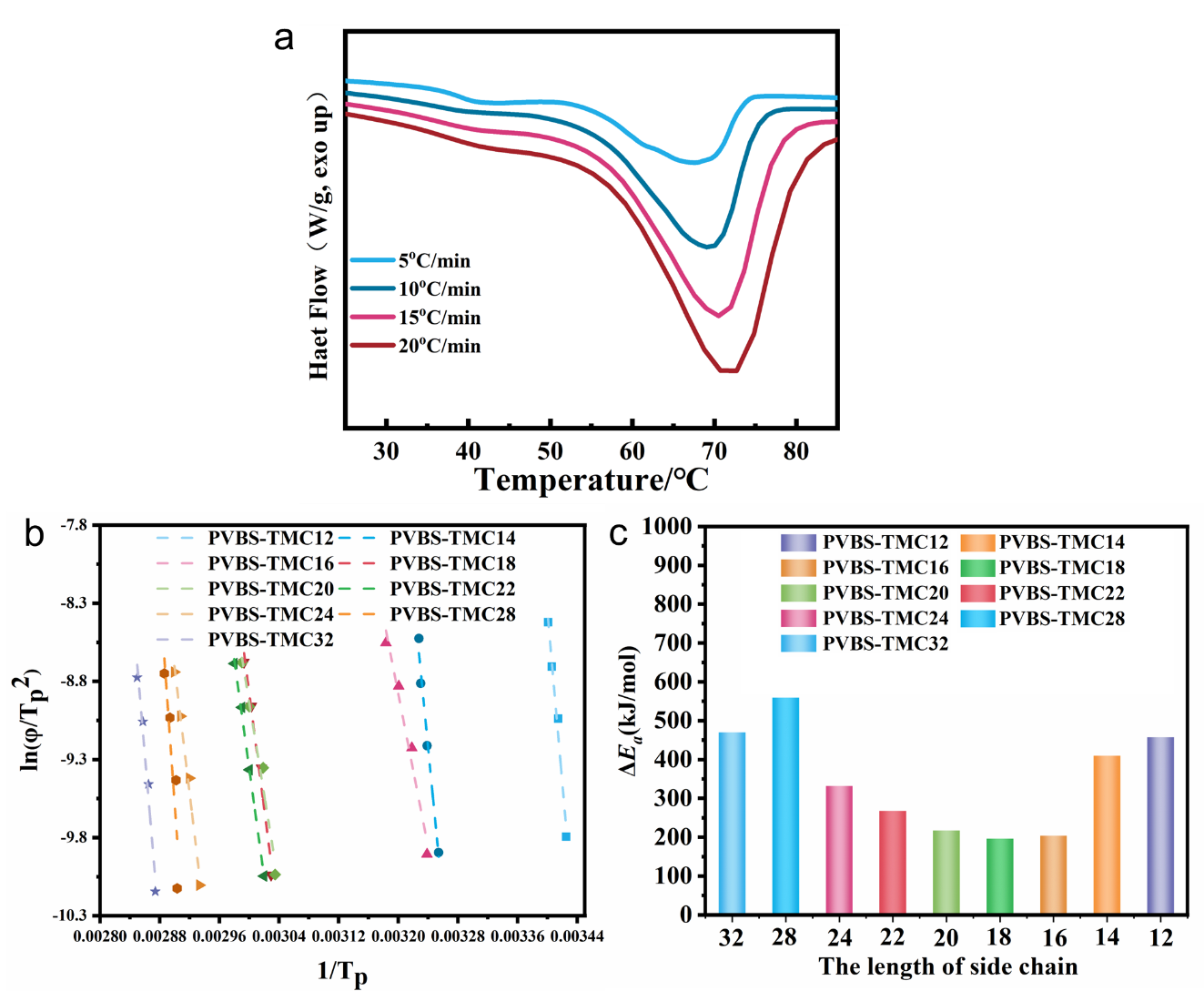
**

**Figure S18.** (a) DSC curves of PVBS-TMC_n_ under different heating rate. (b) The curves of ln ($\varphi$/$T_{p}^{2}$) and 1/$T_{p}$, and (c) the activation energy (Δ*E_a_*) of PVBS-TMC_n_ (n=12, 14, 16, 18, 20, 22, 24, 28 and 32).

The Δ*E*_a_ of nonisothermal phase transition can be calculated by Kissinger’s, where $\varphi$ is the heating or cooling rate, *T*_p_ is the phase change temperature, A is the frequent factor and R is the gas constant. Δ*E*_a_ can be calculated through the slope of the curve of $\ln\left( \frac{\varphi}{T_{p}^{2}} \right)$ against $\frac{1}{T_{P}}$.

$$\ln\left( \frac{\varphi}{T_{p}^{2}} \right)=-\frac{{\Delta E}_{a}}{R}\cdot\frac{1}{T_{P}}+ln(\frac{A}{{\Delta E}_{a}}R)$$

For crystalline bottlebrush polymers, an increase in side chain length typically reduces chain entanglement and facilitates crystallization, leading to a lower activation energy. This trend is indeed observed in our PVBS-TMC_12_ to PVBS-TMC_18_ polymer networks. However, as the chain length increases further, the crystallization activation energy begins to rise. This reversal can be attributed to the forced entanglement within the high-density side-chain double-brush architecture, coupled with the melting point approaching to the exchange temperature of dynamic bonds. These factors collectively promote energy dissipation during the crystallization process, resulting in an increasing trend in activation energy.


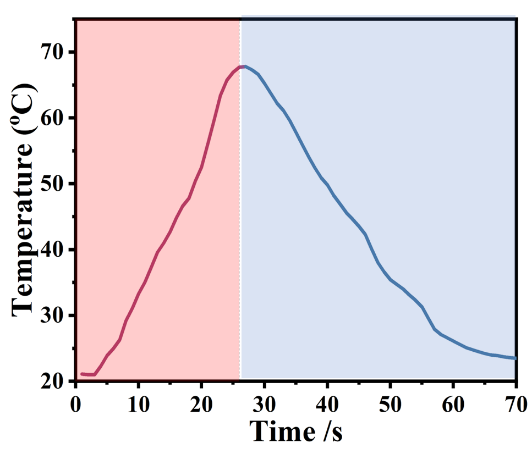


**Figure S19.** The temperature of surface of PVBS-TMC/CNTs under 1W IR irradiation.


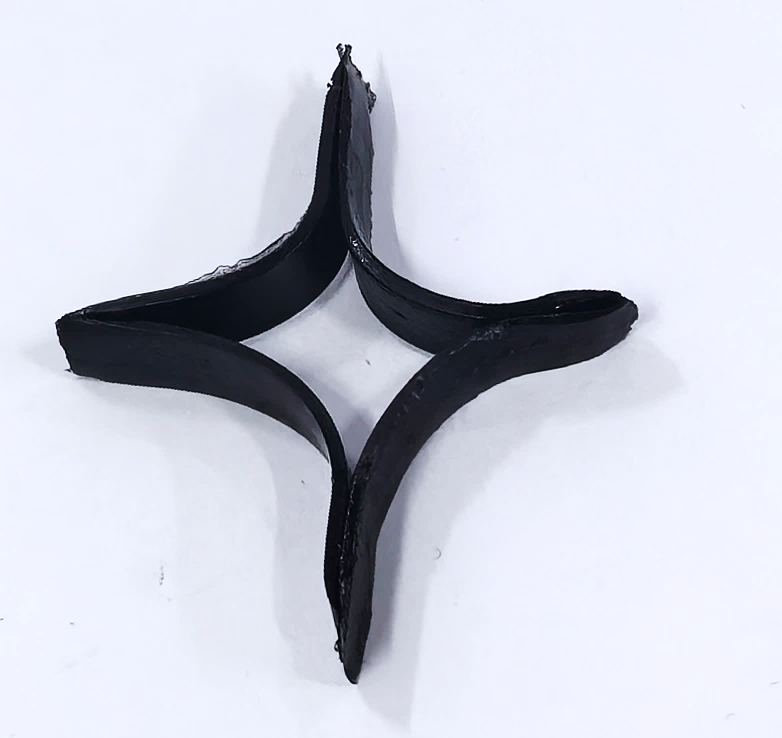


**Figure S20.** The star-shaped PVBS-TMC/CNTs obtained by infrared welding.


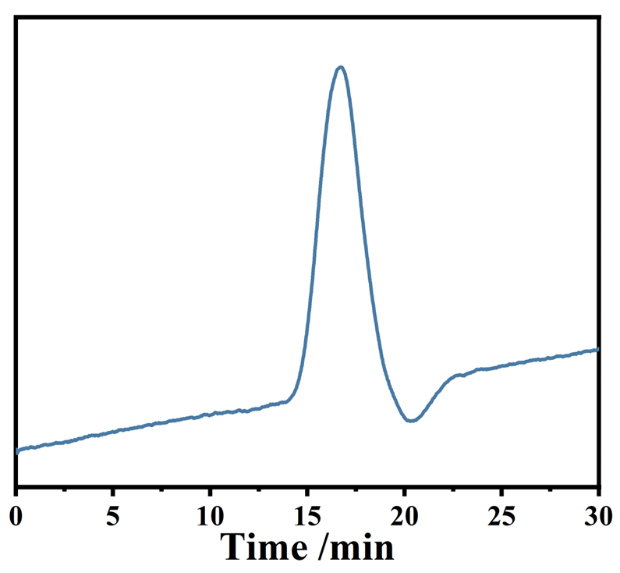


**Figure S21.** The GPC curve of PVBS-TMC_32_ after dissolution in THF/MeOH solvent.


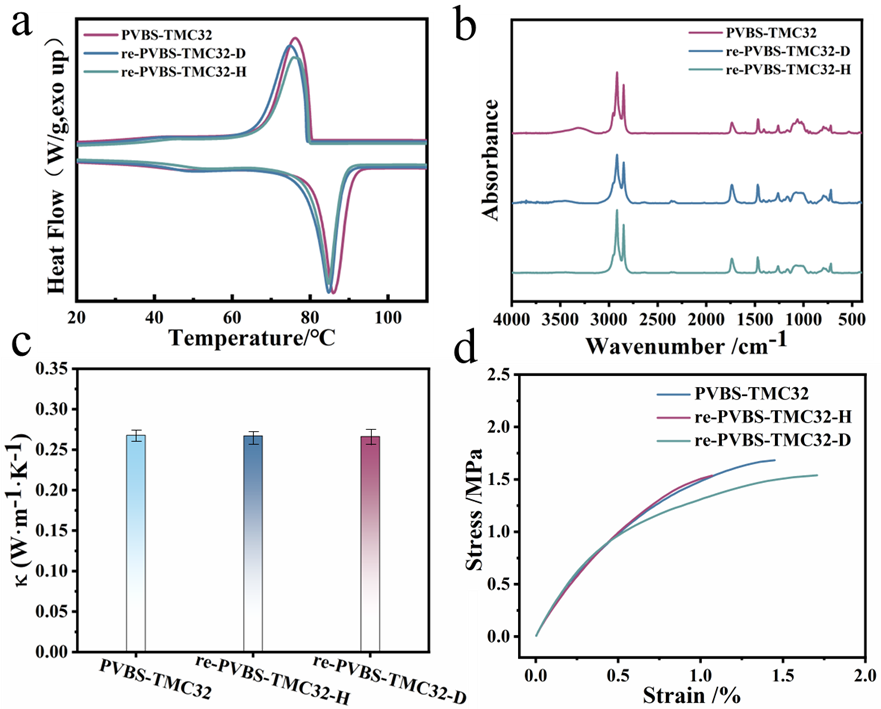


**Figure S22.** (a) DSC and (b) FT-IR spectra, (c) thermal conductivity and (d) tensile strength curve of PVBS-TMC_32_ before and after recovery.


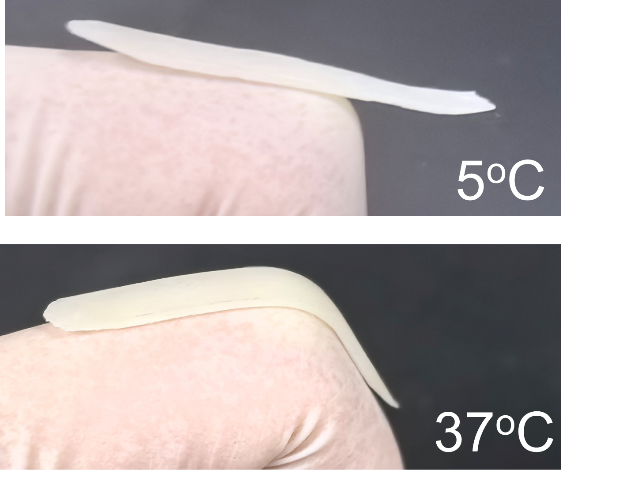


**Figure S23.** Flexibility-rigid switching of PVBS-TMC_14/24_ at 37 ^o^C and 5 ^o^C.


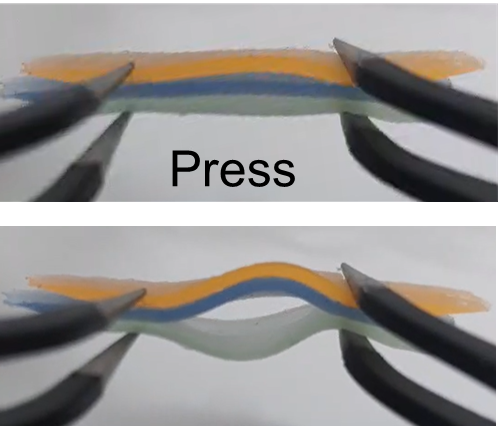


**Figure S24.** Dyeability and flexibility of PVBS-TMC_14/24_.


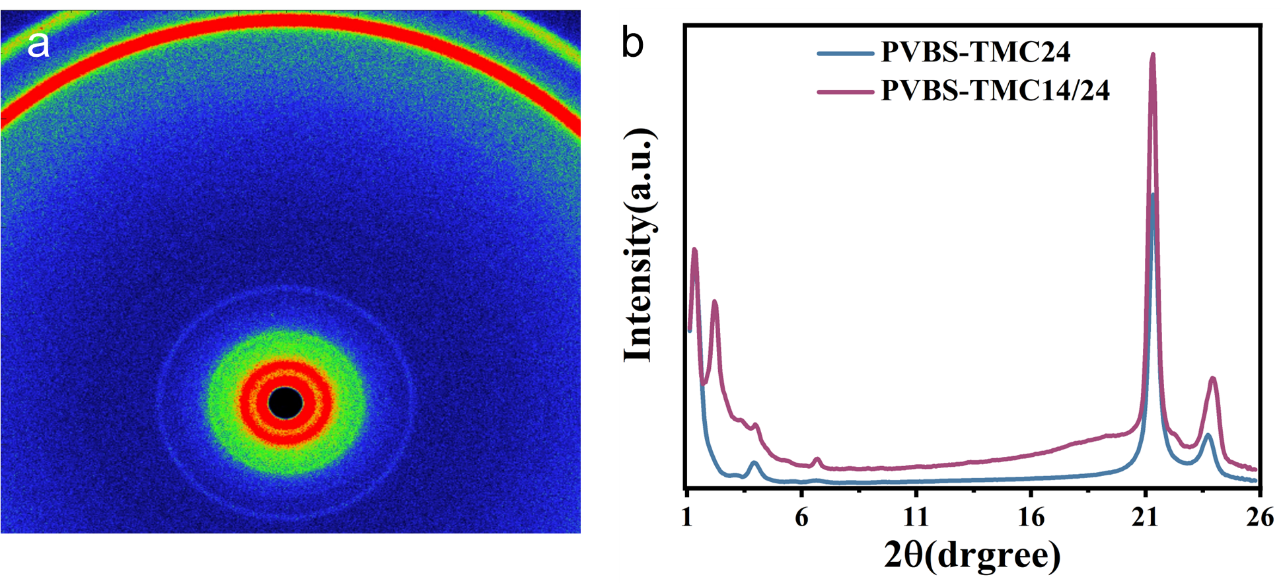


**Figure S25.** (a) The WAXS/SAXS results of PVMS-TMC_24_. (b) The relationship between degree and intensity of PVMS-TMC_24_ and PVBS-TMC_14/24_ at random direction.


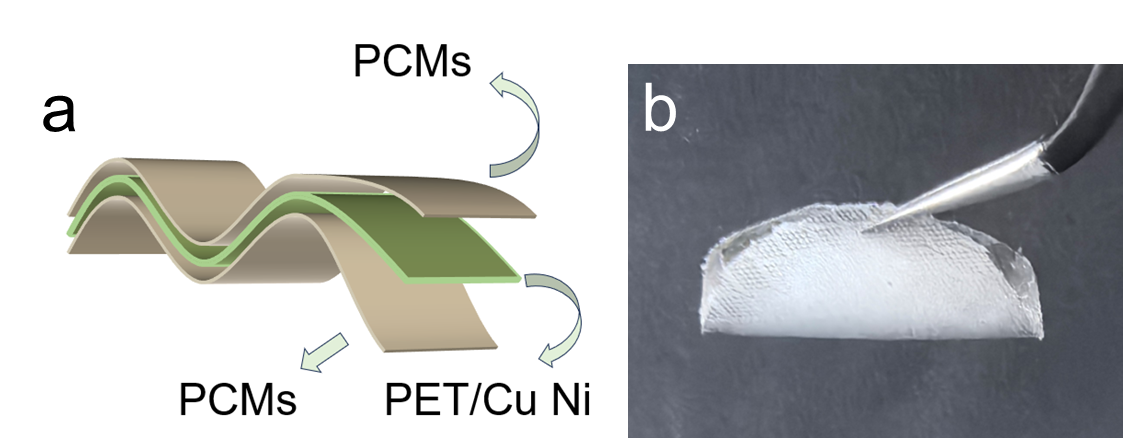


**Figure S26.** (a) Preparation of flexible electromagnetic shielding phase-change composite Cu/Ni-PVBS and (b) visual diagram.


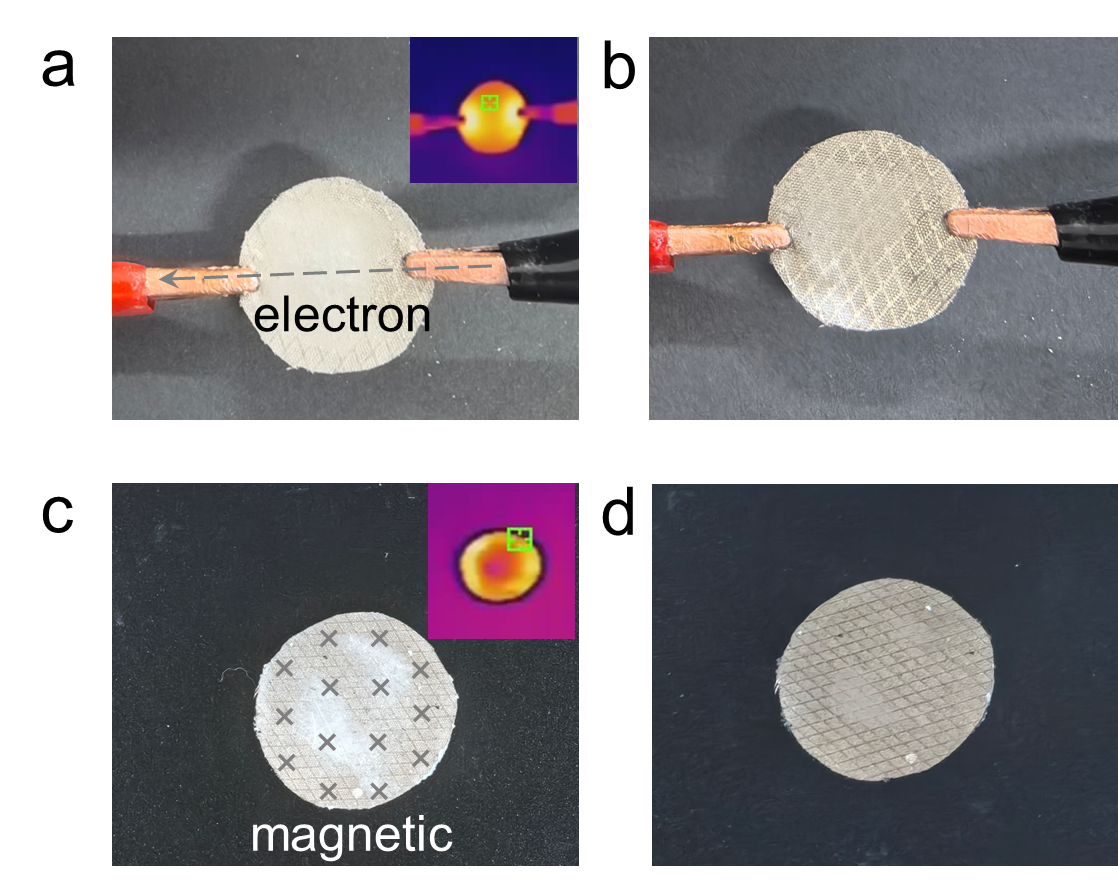


**Figure S27.** (a, b) Electrothermal properties and (c, d) magnetothermal properties of Cu/Ni-PVBS.


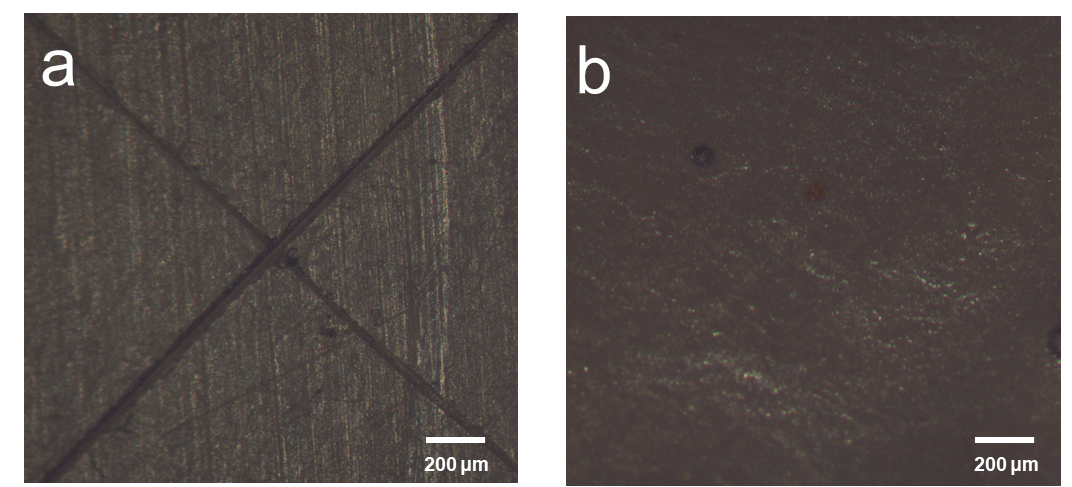


**Figure S28.** (a, b) Self-healing microscope images under heating.


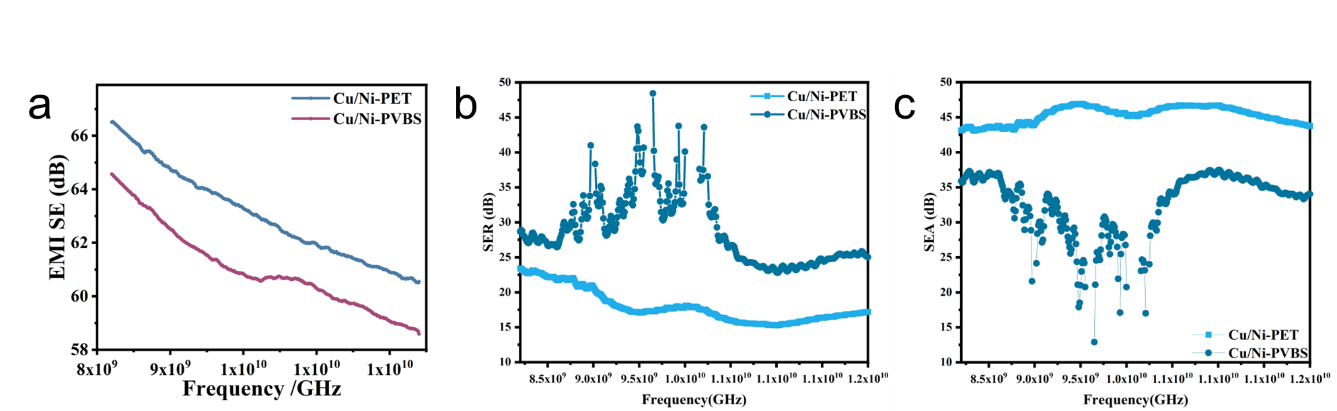


**Figure S29.** (a) EMI SE (b) SEA and (c) SER curve of Cu/Ni-PVBS and Cu/Ni-PET with single layer.


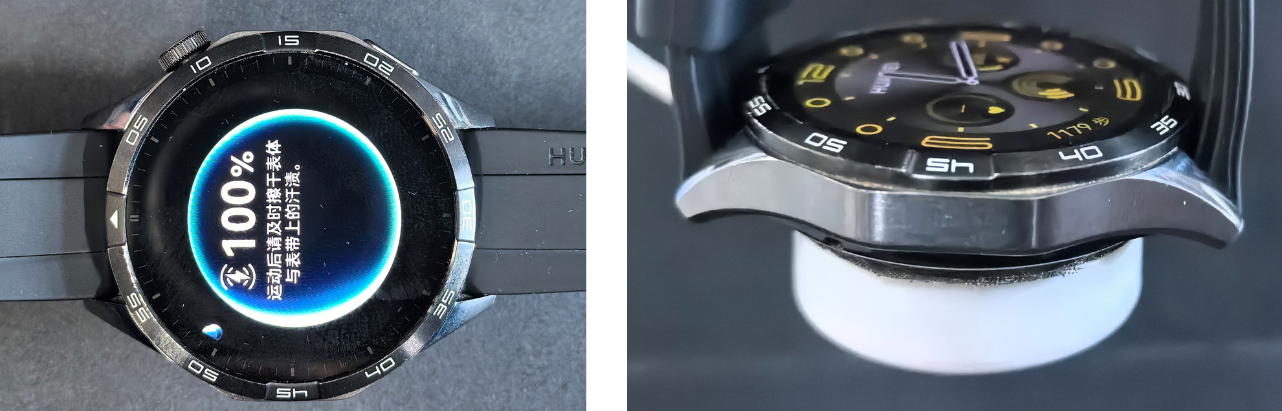


**Figure S30.** Shielded wireless charging behavior of Cu/Ni-PVBS.


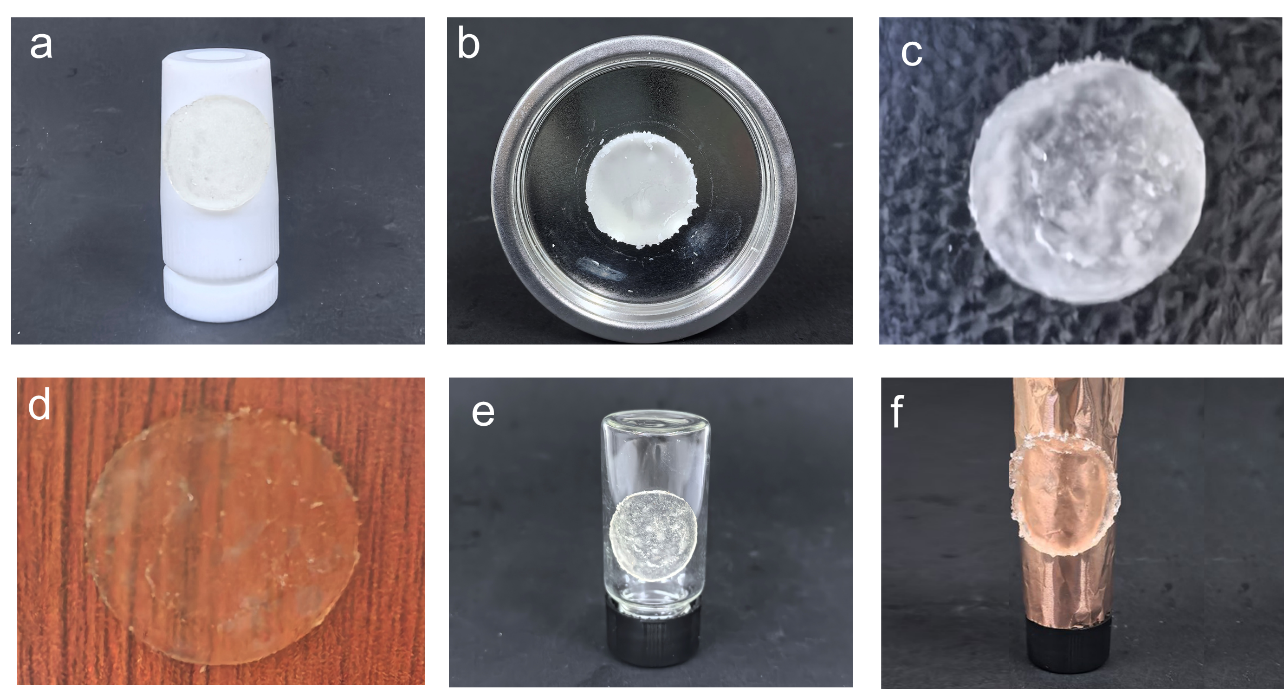


**Figure S31.** The adhesion behavior of PVBS-TMC_n_ on PTFE (a), aluminum (b), leather (c), wood (d), glass (e) and copper (f).


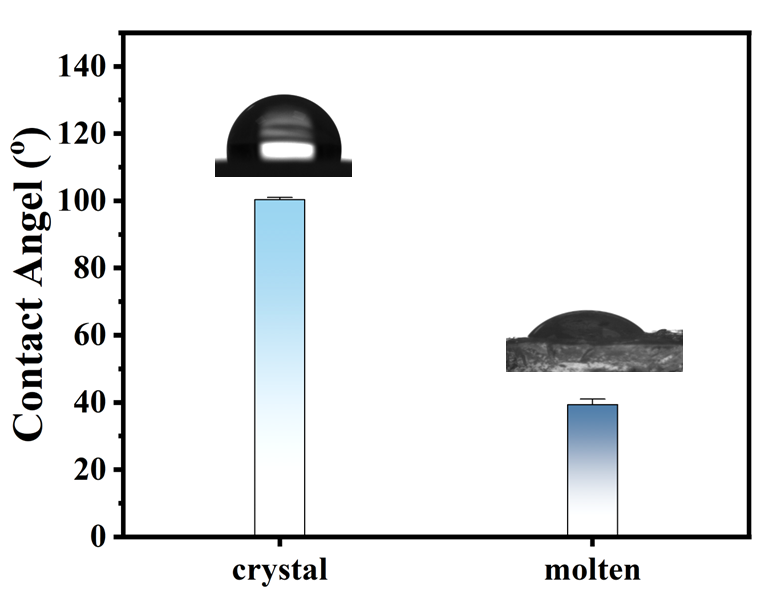


**Figure S32.** Water contact angle of PVBS-TMC_14/24_.


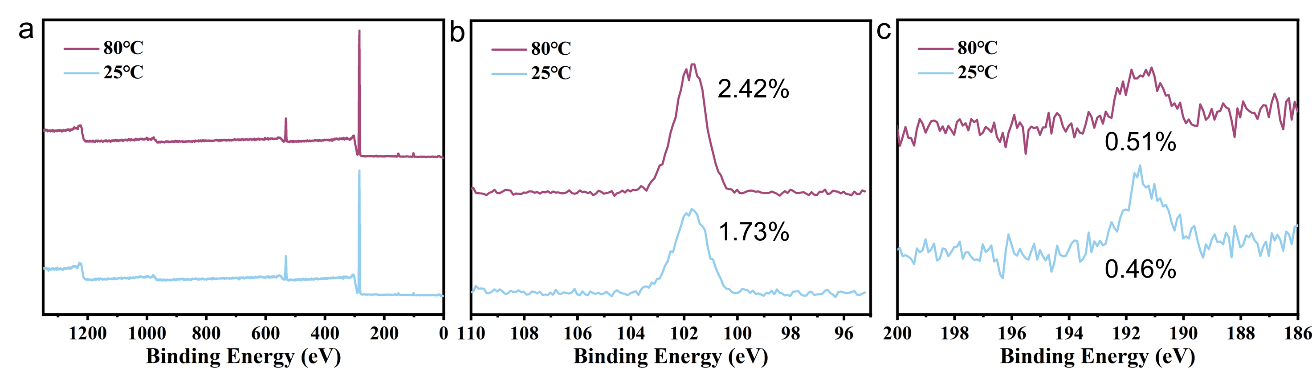


**Figure S33.** Variable temperature XPS full spectra of PVBS-TMC_14/24_ (a). Variable temperature high-resolution XPS Si2s (b) and B1s (c) spectra of PVBS-TMC_14/24_.

Due to the excessive phase-change units, carbon chain, in PVBS-TMC_14/24_, most of the XPS is C-C bonds (≈89%), which makes the signal-to-noise ratio of the remaining elements very poor. However, the surface migration of silicon and boron atoms at the high-temperature air interface can still be measured.


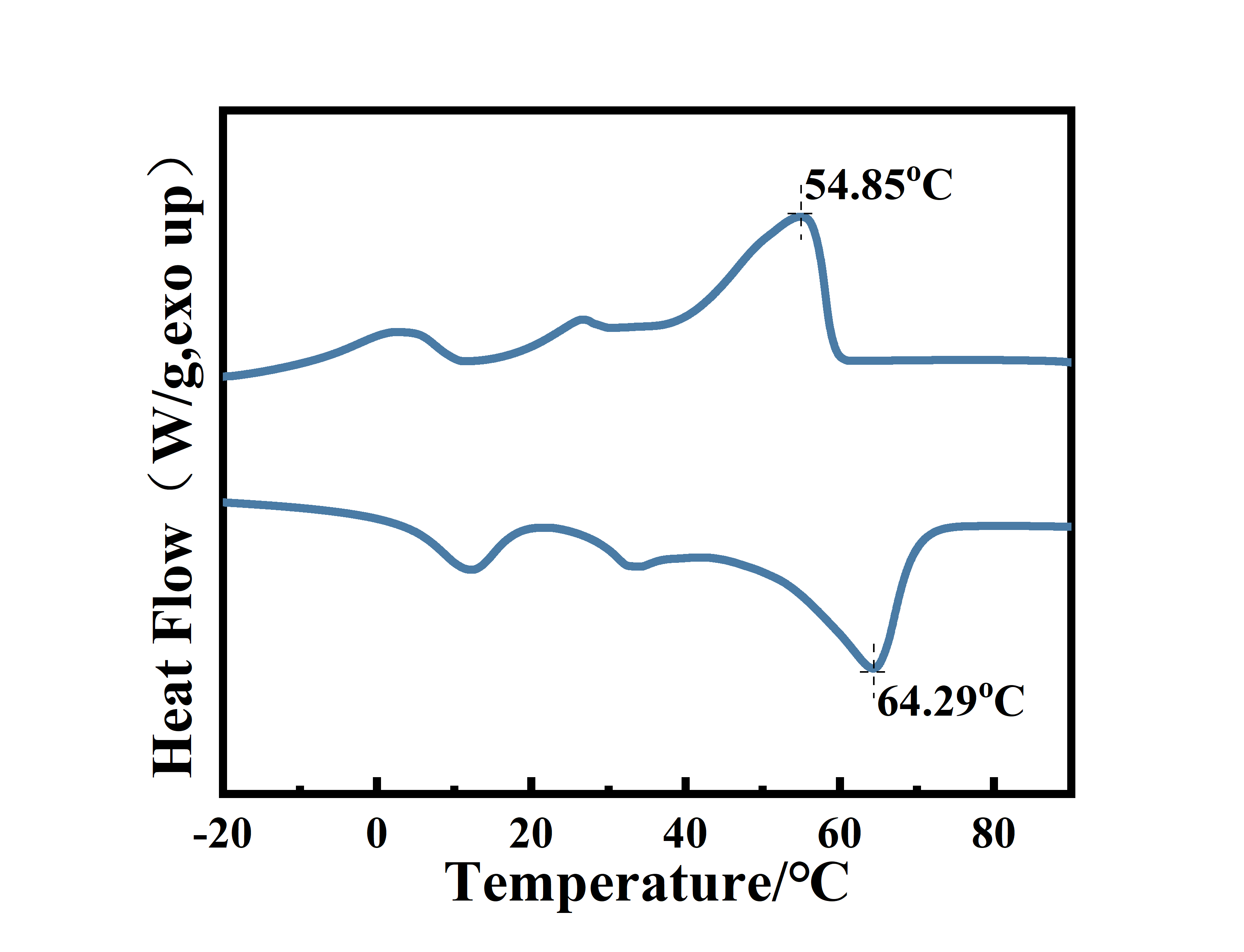


**Figure S34.** The DSC curve of PVBS-TMC/CF.


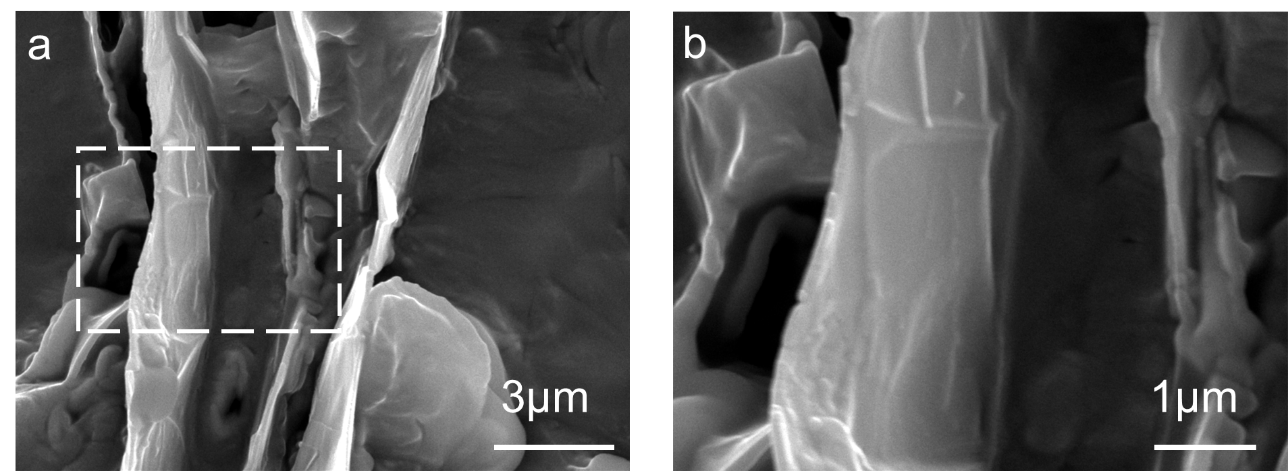


**Figure S35.** SEM images of PVBS-TMC/CF.


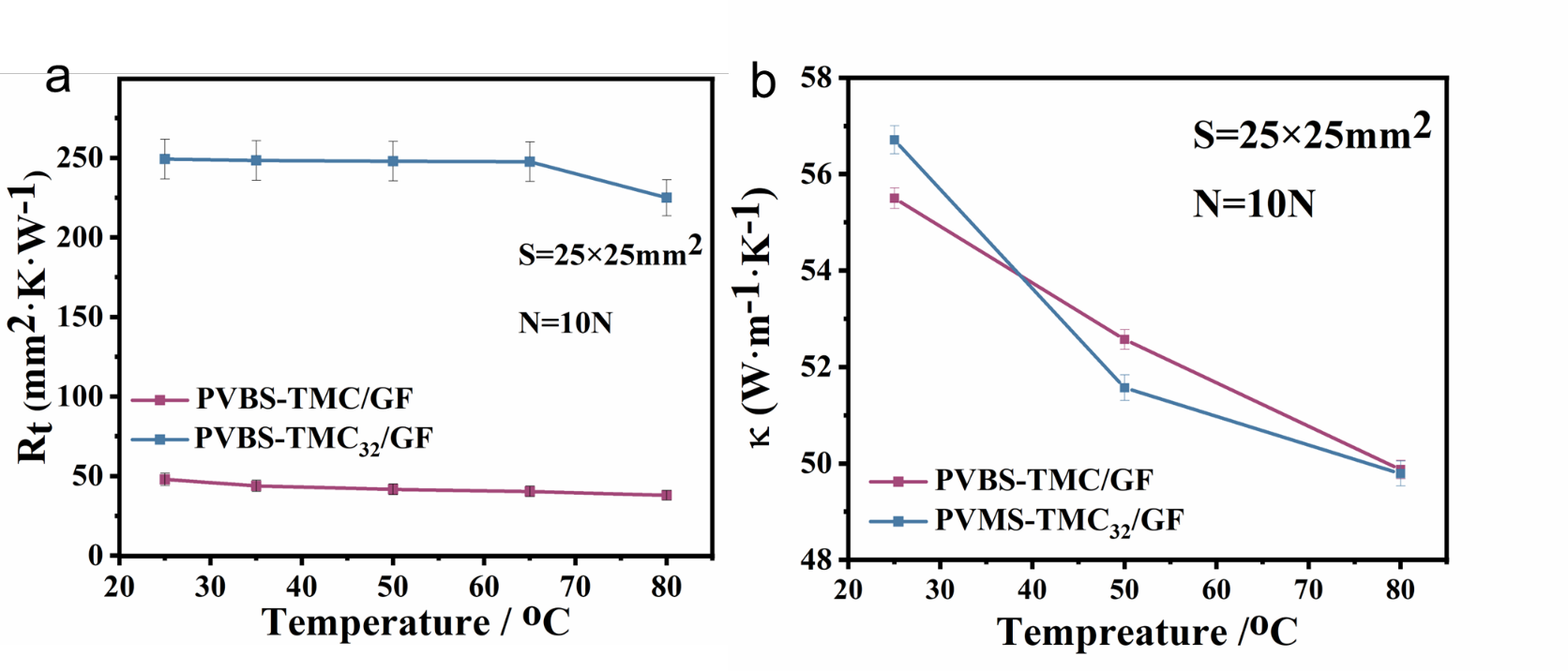


**Figure S36.** (a) ITR and (b) thermal conductivity k under different temperature of PVBS-TMC/CF and PVBS-TMC_32_/CF


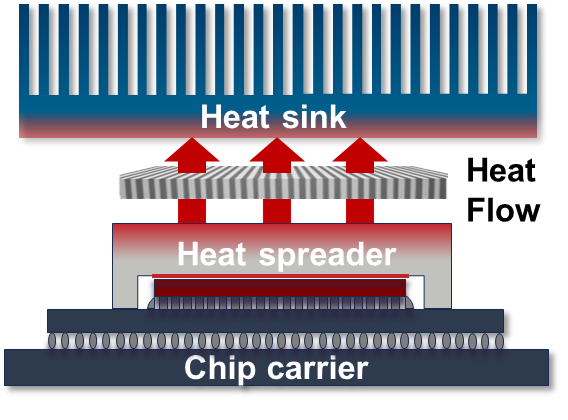


**Figure S37.** Positional relationship of TIM, graphene foam films composite and heat-sink.


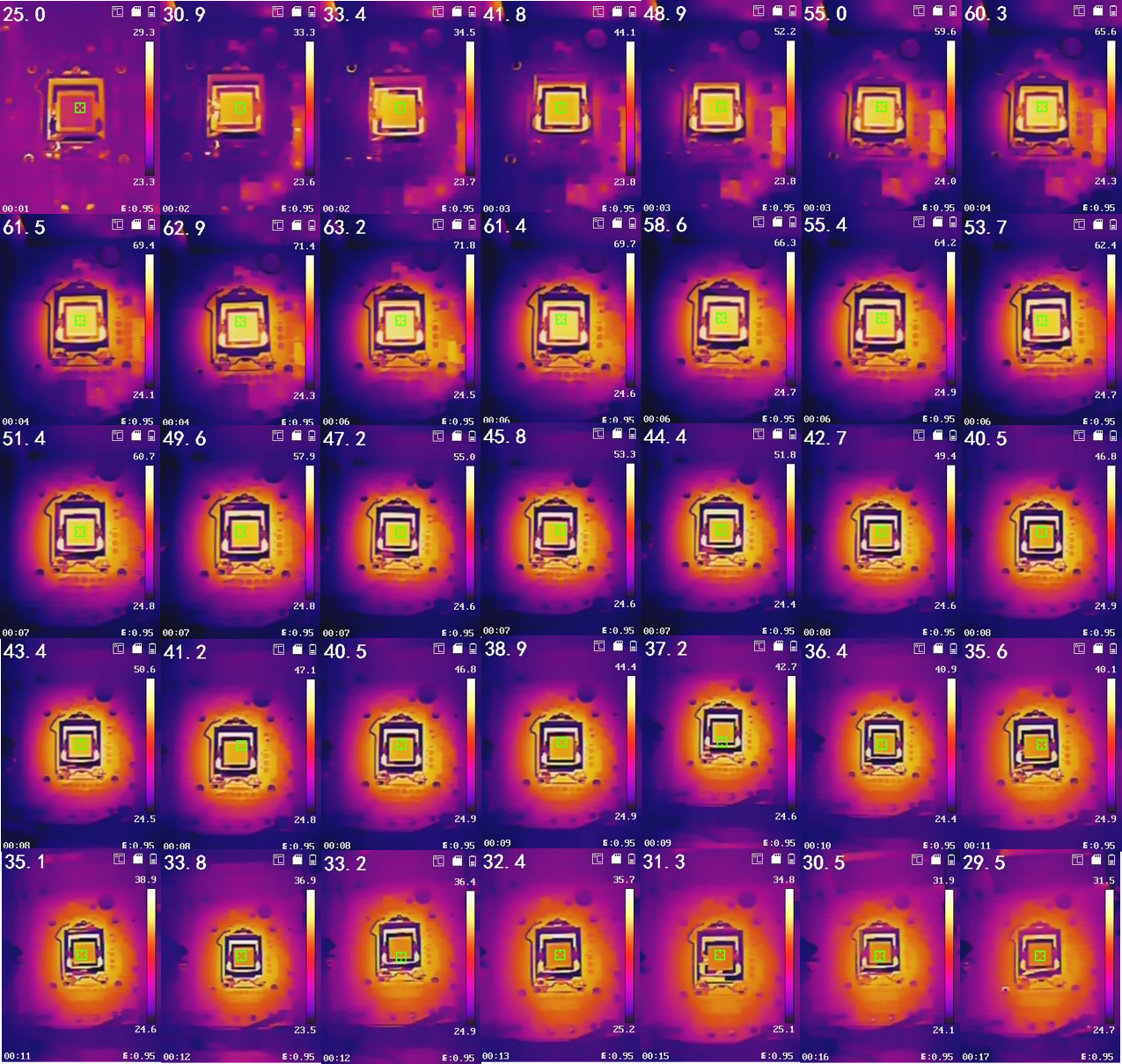


**Figure S38.** Infrared thermal images of PVBS-TMC/CF shot per 10 seconds on CPU without heat sink.


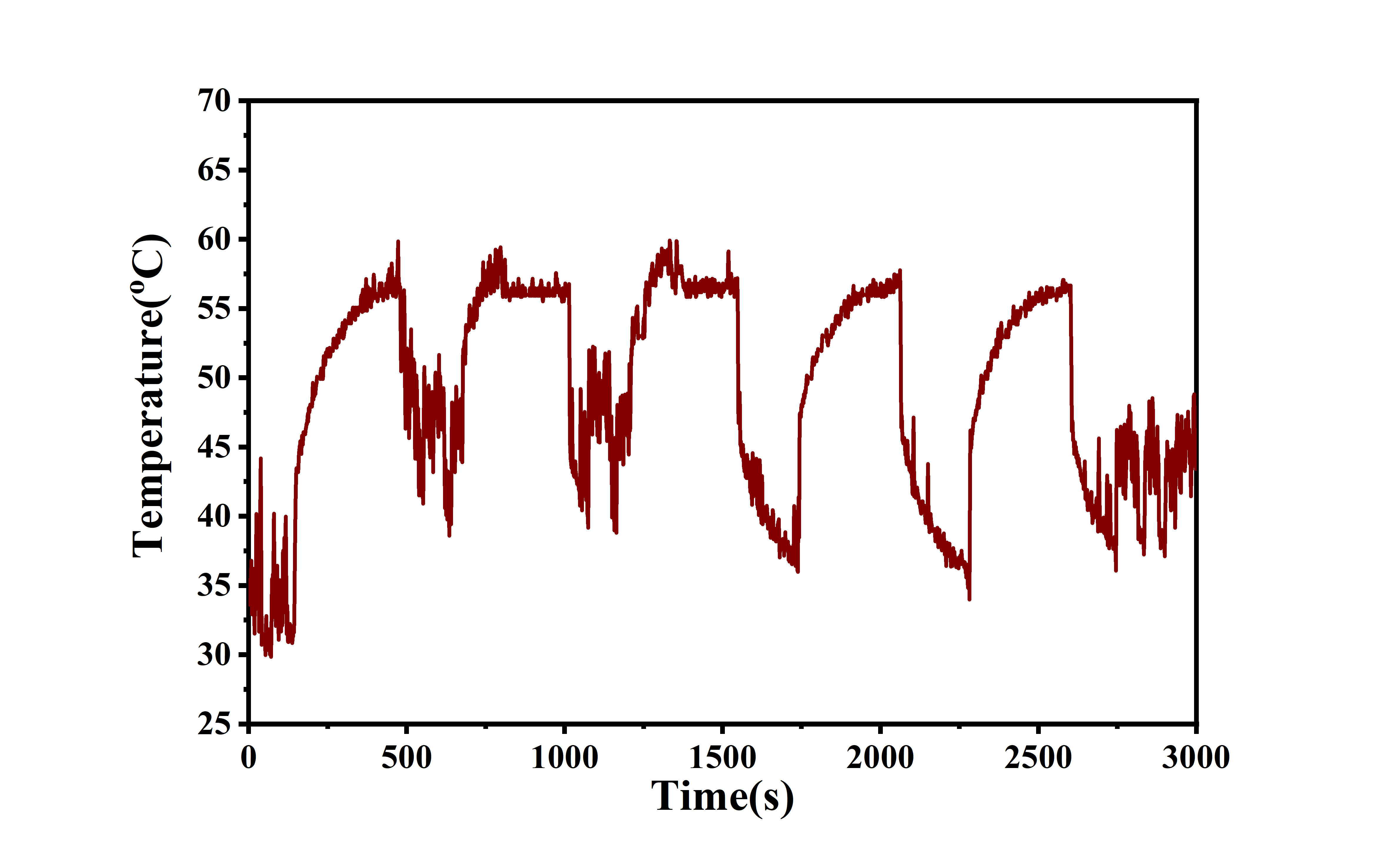


**Figure S39.** Temperature of CPU equipping PVMS-TMC/CF was detected within five heating-cooling cycles.

The absence of dynamic bonds in PVMS-TMC/CF reduces chain mobility, impeding interfacial void filling and leading to premature attainment of peak temperature. Achieving thermal equilibrium with the heat sink requires three operational cycles to establish stable conformal contact. Nevertheless, both the peak and steady-state temperatures remain higher than those of PVBS-TMC/CF.


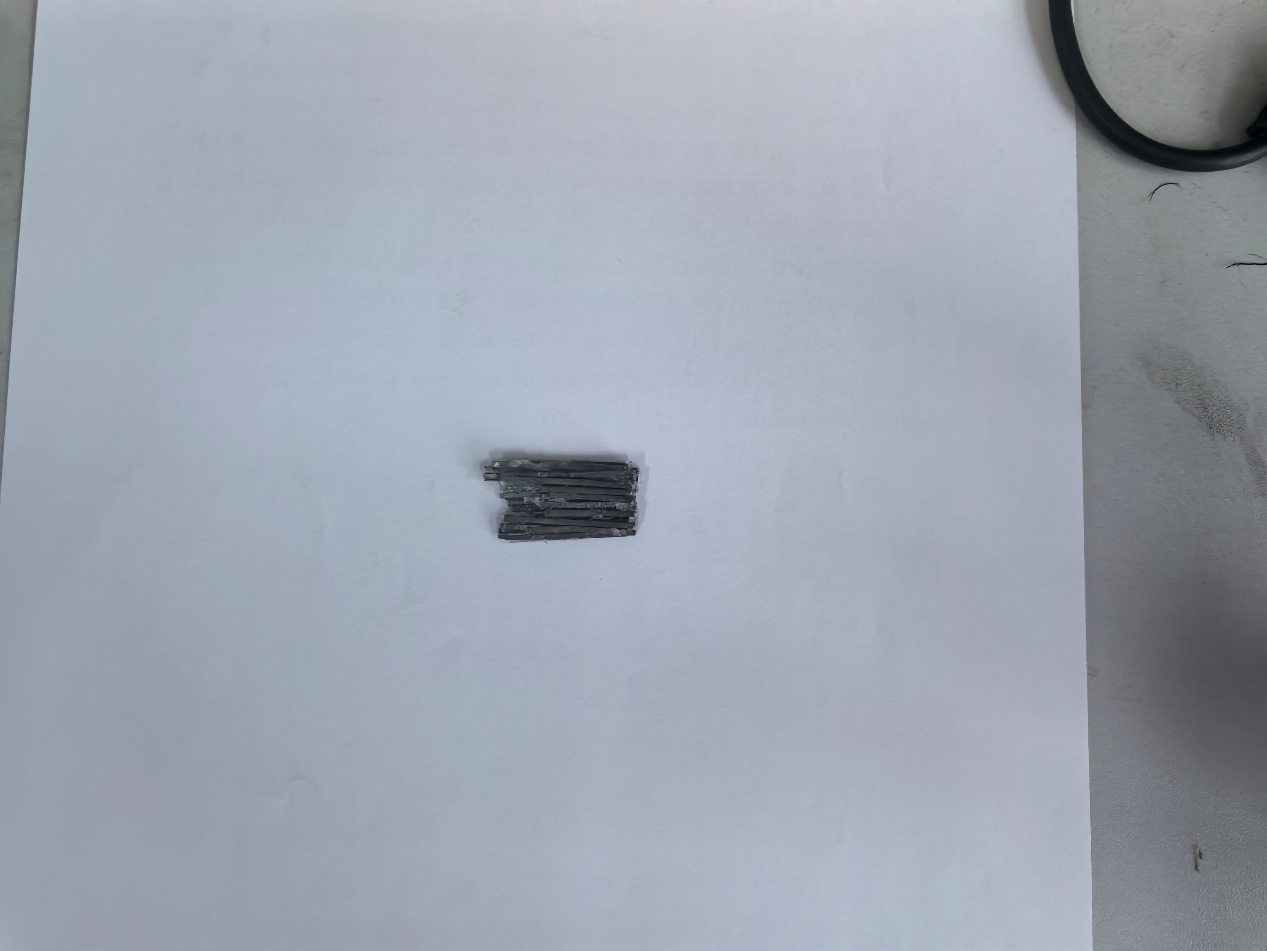


**Figure S40.** The photo of recycled PVBS-TMC/GF.

Supplementary Tables

**Table S1.**

GPC results of PVMS and PVBS.

| Sample | M_n_ (g/mol) | *Đ* |
| --- | --- | --- |
| PVMS | 6300 | 1.74 |
| PVBS | 14000 | 2.99 |

**Table S2.**

Thermal characteristics of 1-n-alkanol, TMC_n_, PVBS-TMC_n_, mainchain and crosslinker.

| Saple | T_m_  (^o^C) | Δ*H*_m_  (J·g⁻¹) | Δ*H*_m_^T^  (J·g⁻¹) | Δ*H*_m loss_  (%) | T_f_ | Δ*H*_f_  (J·g⁻¹) | Δ*H*_f_^T^  (J·g⁻¹) | Δ*H*_f loss_  (%) |
| --- | --- | --- | --- | --- | --- | --- | --- | --- |
| 32OH | 88.00 | 304.4 | / | / | 81.26 | 306.6 | / | / |
| 30OH | 82.62 | 307.8 | / | / | 80.15 | 319.2 | / | / |
| 28OH | 83.38 | 309.5 | / | / | 77.23 | 320.4 | / | / |
| 24OH | 76.85 | 280.9 | / | / | 68.40 | 283.4 | / | / |
| 22OH | 72.04 | 271.2 | / | / | 62.09 | 270.7 | / | / |
| 20OH | 70.02 | 270.6 | / | / | 58.10 | 270.8 | / | / |
| 18OH | 63.78 | 268.5 | / | / | 51.62 | 263.5 | / | / |
| 16OH | 52.07 | 222.8 | / | / | 22.70 | 224.4 | / | / |
| 14OH | 41.10 | 251.8 | / | / | 30.18 | 254.7 | / | / |
| 12OH | 24.97 | 248.2 | / | / | 16.77 | 240.5 | / | / |
| 10OH | 7.67 | 211.8 | / | / | -1.27 | 211.0 | / | / |
| 8OH | -14.43 | 100.0 | / | / | -27.59 | 95.5 | / | / |
| TMC_32_ | 88.01 | 264.0 | / | / | 79.69 | 261.4 | / | / |
| TMC_30_ | 85.36 | 252.1 | / | / | 77.56 | 263.0 | / | / |
| TMC_28_ | 79.66 | 232.5 | / | / | 71.72 | 234.7 | / | / |
| TMC_24_ | 72.46 | 244.2 | / | / | 63.18 | 258.7 | / | / |
| TMC_22_ | 58.55 | 180.8 | / | / | 55.32 | 178.3 | / | / |
| TMC_20_ | 53.80 | 176.2 | / | / | 47.21 | 172.9 | / | / |
| TMC_18_ | 51.92 | 189.5 | / | / | 42.76 | 213.6 | / | / |
| TMC_16_ | 36.38 | 161.2 | / | / | 28.78 | 164.4 | / | / |
| TMC_14_ | 32.62 | 146.3 | / | / | 14.04 | 154.1 | / | / |
| TMC_12_ | 17.49 | 152.7 | / | / | 2.02 | 159.3 | / | / |
| TMC_10_ | -2.53 | 129.6 | / | / | -19.34 | 139.2 | / | / |
| PVBS-TMC_32_ | 83.36 | 240.7±1.8 | 240.8 | 0.06 | 78.58 | 240.2±0.5 | 240.8 | 0.26 |
| PVBS-TMC_30_ | 82.18 | 211.0±2.1 | 225.2 | 5.31 | 77.63 | 213.8±1.7 | 224.3 | 4.89 |
| PVBS-TMC_28_ | 78.61 | 202.9±0.3 | 206.4 | 1.70 | 67.82 | 200.1±0.4 | 208.0 | 3.97 |
| PVBS-TMC_24_ | 74.16 | 166.4±1.5 | 213.5 | 22.08 | 55.63 | 161.7±1.1 | 166.0 | 28.52 |
| PVBS-TMC_22_ | 60.17 | 148.3±1.7 | 156.7 | 0.35 | 52.05 | 143.9±0.9 | 146.6 | 1.90 |
| PVBS-TMC_20_ | 58.12 | 121.2±2.1 | 151.1 | 19.78 | 38.48 | 116.0±1.1 | 148.3 | 21.76 |
| PVBS-TMC_18_ | 58.38 | 157.9±0.2 | 160.5 | 1.64 | 48.41 | 153.0±0.7 | 180.9 | 15.44 |
| PVBS-TMC_16_ | 33.09 | 93.5±0.4 | 134.6 | 30.55 | 23.93 | 92.2±0.4 | 137.3 | 32.85 |
| PVBS-TMC_14_ | 35.94 | 103.9±0.3 | 120.1 | 13.52 | 22.65 | 97.8±0.7 | 126.5 | 22.71 |
| PVBS-TMC_12_ | 19.82 | 65.4±1.1 | 122.9 | 46.77 | 7.34 | 60.3±0.6 | 128.2 | 52.95 |
| PVBS-TMC_10_ | -29.4 | 34.6±0.4 | 101.7 | 65.97 | -39.36 | 36.1±0.3 | 109.2 | 66.95 |
| PVBS-TMC_14/24_ | 64.21 | 140.7±2.3 | / | / | 54.31 | 136.8±2.5 | / | / |
| PVBS-TMC/GF | 64.29 | 99.7 | / | / | 54.85 | 94.4 | / | / |
| PVBS | 42.18 | 2.1 | / | / | 21.09 | 1.9 | / | / |
| PAD | 74.85 | 172.5 | / | / | 68.14 | 164.6 | / | / |
| TSH | -38.73 | 15.4 | / | / | -48.95 | 11.6 | / | / |

Notes: *T*_m_: peak melting temperature, *T*_f_: peak crystallization temperature, Δ*H*_m_: actual melting enthalpy, Δ*H*_f_: actual crystallization enthalpy, Δ*H*^T^: the theoretical enthalpy, Δ*H*_m loss_ (Δ*H*_f loss_) represents the percentage value of the difference between Δ*H*_m_^T^ (Δ*H*_f_^T^) and Δ*H*_m_ (Δ*H*_f_) relative to Δ*H*_m_^T^ (Δ*H*_f_^T^).

**Table S3.**

Comparisons of Δ*H*, Δ*H*_loss_ and *T*_m_ of reported PCPNs.

|  | *T*_m_ (^o^C) | Δ*H*(J·g^-1^) | Δ*H*_loss_(%) |
| --- | --- | --- | --- |
| Ref. S1. | 72.5 | 156.8 | 8.26 |
| Ref. S2. | 52.9 | 156.2 | 3.21 |
| Ref. S3. | 50.47 | 55.75 | 36.36 |
| Ref. S4. | 57.9 | 142.5 | 16.67 |
| Ref. S5. | 57.1 | 128.3 | 19.90 |
| Ref. S6. | 27.33 | 92.01 | 24.44 |
| Ref. S7. | 40.6 | 128 | 21.80 |
| Ref. S8. | 53.6 | 125.3 | 32.20 |

**Table S4.**

Latent heat values of PVBS-TMC_n_ for cycle 1, 10, and 50.

| Saple | cycle 1 | cycle 10 | cycle 50 |
| --- | --- | --- | --- |
| PVBS-TMC_32_ | 240.7 | 240.1 | 238.9 |
| PVBS-TMC_30_ | 211.0 | 210.6 | 209.3 |
| PVBS-TMC_28_ | 202.9 | 202.1 | 199.7 |
| PVBS-TMC_24_ | 166.4 | 165.8 | 164.1 |
| PVBS-TMC_22_ | 148.3 | 148.4 | 148.1 |
| PVBS-TMC_20_ | 121.2 | 120.9 | 120.3 |
| PVBS-TMC_18_ | 157.9 | 157.9 | 158.1 |
| PVBS-TMC_16_ | 93.5 | 93.2 | 92.7 |
| PVBS-TMC_14_ | 103.9 | 103.9 | 101.0 |
| PVBS-TMC_12_ | 65.4 | 64.1 | 60.4 |
| PVBS-TMC_10_ | 34.6 | 33.9 | 31.1 |

Notes: The numbers in the table represent enthalpy values, with the unit being J·g⁻¹.

**Table S5.**

Comparisons of relaxation times of reported CANs.

|  | Relaxation Times t (s) | Relaxation Temperature T (^o^C) |
| --- | --- | --- |
| Ref. S9. | 5 | 60 |
| Ref. S10. | 100 | 180 |
|  | 30 | 140 |
| Ref. S11. | 80 | 80 |
| Ref. S12. | 0.5 | 190 |
| Ref. S13. | 779 | 180 |
| Ref. S14. | 60 | 55 |

**Table S6.**

Crystal characteristics calculated by Bragg formula and Debye-Scherrer formula of PVBS-TMC_24_ and PVBS-TMC_14/24_.

| Sample | Scattering verctor q (Å^-1^) | Incident angle 2q (^o^) | Interplanar spacing d (Å) | Peak width at half-height B (^o^) | Crystal thickness D (Å) |
| --- | --- | --- | --- | --- | --- |
| PVBS-TMC_14/24_ | 0.0119 | 1.3 | 64.8 | 0.48 | 162.1 |
|  | 0.0191 | 2.1 | 40.4 | 0.83 | 95.0 |
|  | 0.1861 | 21.3 | 4.2 | 0.47 | 171.3 |
|  | 0.2086 | 23.9 | 3.7 | 0.89 | 90.7 |
| PVBS-TMC_24_ | 0.0119 | 1.3 | 64.8 | 0.52 | 150.1 |
|  | 0.1861 | 21.3 | 4.2 | 0.48 | 166.9 |
|  | 0.2069 | 23.7 | 3.8 | 0.99 | 80.7 |

**Table S7.**

Comparisons of κ, ITR, Δ*H* and content of PCMs of reported and commercial TIMs.

|  | κ  (W·m^-1^·K^-1^) | ITR  (K·mm^2^·W^-1^) | Δ*H*  (J·g^-1^) | *T*_m_  (^o^C) | content of PCMs (%) |
| --- | --- | --- | --- | --- | --- |
| Ref. S15. | 43.4 | 30.5 | 56 | 51.8 | 51.5 |
| Ref. S16. | 51.55 | 28.6 | 18.8 | 44 | 40 |
| Ref. S17. | 32.86 | - | 158.2 | 148.2 | 70 |
| Ref. S18. | 45.97 | 6.83 | 57 | - | 38.4 |
| Ref. S19. | 3.41 | - | 141.6 | 40.05 | 76.7 |
| HI-FLOW THF 1600G  (Henkel) | 1.6 | 129.0 | - | 55 | - |
| 5500T  (3M) | 2.5 | 20 | - | 50 | - |
| PTM7950  (Honeywell) | 8.5 | 4.0 | - | 45 | - |
| This work | 55.5 | 35.7 | 99.7 | 64.29 | 75 |

References

1. Wu, Y. *et al.* Recyclable Solid-Solid Phase Change Materials with Superior Latent Heat via Reversible Anhydride‐Alcohol Crosslinking for Efficient Thermal Storage. *Adv. Mater.* **36**, 2311717 (2024).
2. Wang, Y. *et al.* Integrating MXene Film with Recyclable Polyethylene Glycol‐co‐Polyphosphazene Copolymer as Solid–Solid Phase Change Material for Versatile Applications. *Small* **20**, 2407626 (2024).
3. Soo, X. Y. D. *et al.* Polyethylene glycol/polylactic acid block co‐polymers as solid–solid phase change materials. *Smart. Mat.* **4**, e1188 (2023).
4. Wang, C. *et al.* Multiple H‐Bonding Cross‐Linked Supramolecular Solid–Solid Phase Change Materials for Thermal Energy Storage and Management. *Adv. Mater.* **36**, 2309723 (2023).
5. Wang, M. & Zhang, Y. J. Thermal and Mechanical Robust Solid‐Solid Phase Change Materials Enabled by Reactive Crosslinkable Poly (Ethylene Glycol) s via Facile Thiol‐Ene Photo‐Click Chemistry. *Adv. Func. Mater.* **35**, 2420042 (2024).
6. Luo, F. *et al.* Tough and sustainable solid–solid phase change materials achieved via reversible crosslinking for thermal management. *Mater. Horiz.* **12**, 4238-4247 (2025)
7. Ma, J. *et al.* Wearable Thermal Energy Storage Polymeric Materials via the Progressive Phase Change Strategy of Crystalline Bottlebrush Polysiloxane Networks. *Chem. Mater.* **37**, 2546-2560 (2025)
8. Ma, J. *et al.* 3D Printable, Recyclable and Adjustable Comb/Bottlebrush Phase Change Polysiloxane Networks toward Sustainable Thermal Energy Storage. *Energy Storage Mater.* **39**, 294-304 (2021).
9. Chen, M. *et al.* SMART Silly Putty: Stretchable, Malleable, Adherable, Reusable, and Tear‐Resistible Hydrogels. *Small* **19**, 2205854 (2022).
10. D’Ambra, C. A. *et al.* Facile Preparation of Tunable Polyborosiloxane Networks via Hydrosilylation. *Chem. Mater.* **36**, 5935-5942 (2024).
11. Ogden, W. A. & Guan, Z. Recyclable, Strong, and Highly Malleable Thermosets Based on Boroxine Networks. *J. Am. Chem. Soc.* **140**, 6217-6220 (2018).
12. Röttger, M. *et al.* High-performance vitrimers from commodity thermoplastics through dioxaborolane metathesis. *Science* **356**, 62-65 (2017).
13. Nishimura, Y. *et al.* Silyl Ether as a Robust and Thermally Stable Dynamic Covalent Motif for Malleable Polymer Design. *J. Am. Chem. Soc.* **139**, 14881-14884 (2017).
14. Cromwell, O. R. *et al.* Malleable and Self-Healing Covalent Polymer Networks through Tunable Dynamic Boronic Ester Bonds. *J. Am. Chem. Soc.* **137**, 6492-6495 (2015).
15. Abdul Jaleel, *et al.* Covalently Functionalized Leakage‐Free Healable Phase‐Change Interface Materials with Extraordinary High‐Thermal Conductivity and Low‐Thermal Resistance. *Adv. Mater.* **35**, 2300956 (2023).
16. Kang, L. *et al.* Phase change composites with ultra-high through-plane thermal conductivity achieved by vertically-aligned graphite film and double-shelled microcapsules. *Compos. Part A Appl. Sci. Manuf.* **182**, 108162 (2024).
17. Zhao, Z. *et al.* Carbon-based phase change composites with directional high thermal conductivity for interface thermal management. *Chem. Eng. J.* **496**, 154305(2024).
18. Zhang, Y. *et al.* Thermal Interface Engineering in a 3D-Structured Carbon Framework for a Phase-Change Composite with High Thermal Conductivity. *Acs. Appl. Mater. Inter.* **15**, 48235-48245 (2023).
19. Lu, Y. *et al.* Magnetically tightened form-stable phase change materials with modular assembly and geometric conformality features. *Nat. Commun.* **13**, 1397 (2022).
